# Synergistic effects of *Candida albicans* and *Porphyromonas gingivalis* biofilms on epithelial barrier function in a 3D aspiration pneumonia model

**DOI:** 10.3389/fcimb.2025.1552395

**Published:** 2025-03-07

**Authors:** Ewelina Wronowska, Ibeth Guevara-Lora, Aleksandra Brankiewicz, Grazyna Bras, Marcin Zawrotniak, Dorota Satala, Justyna Karkowska-Kuleta, Joanna Budziaszek, Joanna Koziel, Maria Rapala-Kozik

**Affiliations:** ^1^ Department of Comparative Biochemistry and Bioanalytics, Faculty of Biochemistry, Biophysics and Biotechnology, Jagiellonian University in Krakow, Krakow, Poland; ^2^ Doctoral School of Exact and Natural Sciences, Faculty of Biochemistry, Biophysics and Biotechnology, Jagiellonian University, Kraków, Poland; ^3^ Department of Microbiology, Faculty of Biochemistry, Biophysics and Biotechnology, Jagiellonian University, Kraków, Poland

**Keywords:** *Candida albicans*, *Porphyromonas gingivalis*, polymicrobial biofilms, 3D model of epithelial cells, epithelial barrier integrity, aspiration pneumonia, air-liquid interface (ALI) model, tight junctions

## Abstract

**Introduction:**

Polymicrobial infections involving *Candida albicans* and *Porphyromonas gingivalis* represent a significant challenge in maintaining epithelial barrier integrity. This study explores their synergistic effects on epithelial cells using an air-liquid interface (ALI) model.

**Methods:**

Mixed-species biofilms were developed and analyzed for their impact on epithelial permeability and tight junction proteins. The effects of biofilm supernatants on IL-8 secretion and oxidative stress markers were also evaluated. The role of *P. gingivalis* proteases was assessed using a gingipain-null mutant (ΔKΔRAB) compared to the wild-type strain (W83). Survival experiments were conducted using *Galleria mellonella* larvae to examine the pathogenicity of dual-species biofilms.

**Results:**

Mixed-species biofilms significantly increased epithelial permeability and disrupted tight junction proteins, as evidenced by reduced levels of ZO-1 and E-cadherin. These changes were accompanied by oxidative stress, characterized by decreased HO-1 expression and enhanced Bax/Bcl-xL ratios, indicating increased pro-apoptotic activity. Supernatants from dual-species biofilms demonstrated a pronounced effect on epithelial cells, modulating IL-8 secretion and exacerbating oxidative damage. *C. albicans* was identified as the dominant driver of pro-inflammatory responses, while *P. gingivalis* contributed through immune modulation and enzymatic activity, primarily via gingipains. The ΔKΔRAB mutant biofilms caused less epithelial disruption and oxidative stress compared to the wild-type, highlighting the critical role of gingipains in pathogenesis.

**Discussion:**

Survival experiments using *Galleria mellonella* larvae supported these findings, highlighting the reduced survival associated with dual-species biofilms and the potential for high-dose antimicrobial therapies to mitigate this effect. These results emphasize the cooperative mechanisms of *C. albicans* and *P. gingivalis* in compromising epithelial barriers and underline the importance of combination therapies targeting both fungal and bacterial components in polymicrobial infections.

## 1 Introduction

Aspiration pneumonia (AP) is a pulmonary infection caused by the inhalation of oropharyngeal or gastric contents into the otherwise sterile lower respiratory tract. It predominantly affects vulnerable populations, such as older adults, individuals with impaired swallowing, immunocompromised patients, and is associated with high morbidity and mortality, emphasizing the need for early diagnosis and targeted prevention (Neill and Dean, 2019).

A key risk factor is the degree of bacterial colonization in oral secretions, where even minimal aspiration can initiate infection with a high bacterial load. Also, microaspirations, often undetected, can recur over time, progressively damaging the pulmonary epithelium and weakening defense mechanisms, such as mucociliary transport and macrophage clearance. This increases susceptibility to infection, especially in immunocompromised individuals, where exposure to pathogens in the alveolar space leads to rapid disease progression. Advanced age correlates strongly with aspiration pneumonia, as many older adults experience silent microaspirations that may not be clinically apparent (Simpson et al., 2023). Among patients aged 70 or older hospitalized with pneumonia, dysphagia prevalence was reported at 91.7%, with silent aspirations occurring in over 50% of cases (Almirall et al., 2021).

AP is frequently polymicrobial, with multiple pathogens acting synergistically to exacerbate infection severity. The most frequently isolated bacteria include Gram-negative aerobic and facultative anaerobic bacteria: *Streptococcus pneumoniae, Streptococcus anginosus, Staphylococcus aureus, Klebsiella pneumoniae, Escherichia coli*. *Streptococcus pneumoniae* is particularly notable. Anaerobic bacteria are infrequent causative pathogens of AP but are commonly implicated due to their abundance in the oral cavity. Key species include: *Prevotella* spp.*, Porphyromonas gingivalis, Fusobacterium nucleatum, Bacteroides fragilis*. These bacteria contribute to pneumonia by forming biofilms and producing virulence factors that damage lung tissue, enhancing bacterial persistence and resistance to immune responses or antibiotics (Benedyk et al., 2016; Kamio et al., 2021; Takahashi et al., 2023). *P. gingivalis* is a multifaceted pathogen, predominantly associated with chronic periodontitis, also contributing to various systemic diseases, such as atherosclerosis and coronary artery disease, where *P. gingivalis* virulence factors can induce inflammatory responses and endothelial dysfunction, contributing to plaque formation (Zhang et al., 2021). In rheumatoid arthritis, *P. gingivalis* can use produced peptidyl-arginine deiminase, for host protein citrullination, potentially triggering autoimmune responses characteristic (Wegner et al., 2010). Also, DNA of *P. gingivalis* was detected in the brains of Alzheimer’s patients, suggesting its possible role in neurodegeneration (Dominy et al., 2019). As AP involves the inhalation of oropharyngeal contents, *P. gingivalis* can be also introducing into the lower respiratory tract (Benedyk et al., 2016) where its proteolytic enzymes, particularly gingipains, can disrupt pulmonary epithelial barriers and modulate immune responses, facilitating infection. Many researches indicate that gingipains are critical factors in the development of diseases caused by *P. gingivalis* (Cutler et al., 1995; Lamont and Jenkinson, 1998; Socransky et al., 1998; Hamada et al., 1998; Amano et al., 2010), although its virulence is attributed to several other factors, including fimbriae, lipopolysaccharides (LPS), hemagglutinins, and capsular polysaccharides (O’Brien-Simpson et al., 2001; Bostanci and Belibasakis, 2012; De Diego et al., 2014).

Gingipains belong to cysteine proteases, comprising arginine-specific (RgpA, RgpB) and lysine-specific (Kgp) enzymes (Travis et al., 1997), and are pivotal in degrading host proteins, modulating immune responses, and facilitating nutrient acquisition (Potempa et al., 2005). They can cleave defensins (Carlisle et al., 2009), immunoglobulins, cytokines and various components of the complement cascade (Potempa and Potempa, 2012; Hajishengallis and Lamont, 2014), undermining host defense mechanisms and promoting inflammation. *P. gingivalis* also expresses long (FimA) and short (Mfa1) fimbriae that mediate adhesion to host tissues and other microorganisms, enhancing colonization and biofilm formation (Kuboniwa et al., 2009). These structures are crucial for the bacterium’s invasive capabilities and its ability to evade the host immune system. *P. gingivalis* can also use LPS to modulate host immune responses, sometimes evading detection or triggering chronic inflammation, contributing to tissue destruction (Nativel et al., 2017). The capsule aids in resisting phagocytosis and complement-mediated killing, enhancing bacterial survival within the host (Singh et al., 2011).

Although bacteria constitute a significant part of the oral microflora, the oral cavity is also colonized by yeasts, particularly *Candida albicans. C. albicans* is a yeast-like fungus that commensally colonizes human mucosal surfaces (Huffnagle and Noverr, 2013; Singh et al., 2014) and is harmless to the host as long as its immune system is functioning properly (Gow and Yadav, 2017; Nikou et al., 2019). However, in immunocompromised patients due to lesions, the balance between host and fungal cells is disrupted, which can lead to the development of disseminated life-threatening infections (Perlroth et al., 2007; Sardi et al., 2013 *). C. albicans* is frequently isolated from respiratory samples (e.g., bronchoalveolar lavage), especially in immunocompromised individuals on prolonged mechanical ventilation, or patients receiving broad-spectrum antibiotics, or those with poor oral hygiene (Zarrinfar et al., 2016). Additionally, *C. albicans* has been isolated from root canals and deep periodontal pockets (Feller et al., 2014; O’Donnell et al., 2015; Gomes et al., 2017). The most important virulence factors of *C. albicans* include: (i) the ability to exist in two morphologically distinct forms, such as yeast-like cells and hypha-forming cells, which is crucial for colonizing new niches (Chow et al., 2021; Alonso-Monge et al., 2024); (ii) the production of aspartyl protases (Sap), which can impair the function of host immune proteins (Kaminishi et al., 1995; Naglik et al., 2003); and (iii) the secretion of candidalysin, a fungal cytolytic peptide toxin, that plays a significant role in epithelial membrane damage (Moyes et al., 2016).

Although *Candida* alone rarely causes aspiration pneumonia in immunocompetent individuals, several mechanisms suggest its indirect or contributory role in enhancing of bacterial virulence through the formation of mixed-species (heterotypic) biofilms with oral bacteria. *C. albicans* has been shown to interact synergistically with bacteria to exacerbate disease, via their combined virulence factors. These mechanisms include enhancement bacterial adhesion, where *C. albicans* hyphae serve as a scaffold for bacterial attachment and colonization; biofilm formation, which increases the resistance of both *Candida* and oral bacteria to phagocytosis and antimicrobial treatments; and modulation of bacterial virulence factors, promoting *C. albicans* growth or biofilm formation (Ponde et al., 2021; Satala et al., 2022).

The interaction between *P. gingivalis* and *C. albicans* underscores the critical role of microbial synergy in enhancing the survival, virulence, and colonization potential of anaerobic pathogens. Within this heterotypic biofilm, the fungal component rapidly proliferates under aerobic conditions, consuming oxygen and thereby creating a hypoxic microenvironment conducive to *P. gingivalis* survival and growth (Fox et al., 2014; de Jongh et al., 2024). This biofilm is stabilized by the specific adherence of bacterial cells to fungal hyphae, mediated by the fungal adhesins Als1 and Als3 (Bartnicka et al., 2019; de Jongh et al., 2024; Sztukowska et al., 2018). This interaction promotes the production of gingipains, which facilitate tissue invasion and bloodstream infiltration (Karkowska-Kuleta et al., 2018). Additionally, the fungal filamentous cells and biofilm matrix reduce the susceptibility of both microorganisms to antibiotics and certain antimycotics. However, the two species also compete for essential environmental resources, such as heme, which can influence the morphology and behavior of *P. gingivalis* cells.

While the study provides valuable insights into this synergistic relationship, further research is required to confirm these findings *in vivo* and to better understand their implications for oral health and treatment strategies. The potential for these pathogens to cohabit the same infectious niche is particularly noteworthy when considering their mutual effects on the development of upper and lower respiratory tract diseases (Tamai et al., 2011; Bartnicka et al., 2019), which constitutes the focus of the present study.

The research was conducted using human bronchial epithelium cultured at the air-liquid interface (ALI). ALI cultures enable the development of 3D cellular models in which basal cells are in contact with the culture medium, while cells on the apical side are exposed to air (Lacroix et al., 2018). Differentiated, pseudostratified human airway epithelium exhibits properties characteristic of *in vivo* cells, such as increased production of adhesion markers (e.g., E-cadherin), tight junction proteins (e.g., ZO-1), formation of ciliated cells, and mucin secretion by goblet cells (Kikuchi et al., 2004; Stewart et al., 2012).

The objective of this study was to investigate the ability of *Candida albicans* and *Porphyromonas gingivalis* to form mixed biofilms on human airway epithelium and to evaluate their impact on epithelial barrier integrity and host cellular response. Specifically, we aimed to analyze the effects of direct contact with these pathogens during infection, as well as the impact of secreted factors from the mixed biofilm, on epithelial cell viability, permeability, and inflammatory response (IL-8 production). To identify the contribution of bacterial gingipains and fungal factors to epithelial barrier disruption, we employed a 3D lung model, assessing both wild-type and gingipain-mutant strains of *P. gingivalis*. Our findings highlight the protective role of *C. albicans* in relation to bacterial activity, suggesting new mechanisms of microbial cooperation and potential therapeutic targets for addressing epithelial stress and barrier dysfunction.

## 2 Materials and methods

### 2.1 Cell culture

Human bronchial epithelial BEAS-2B cells (CRL-3588, ATCC, Manassas, VA, USA) were cultured in serum- and gentamicin-free grown factor-supplemented medium (BEGM, Lonza, Basel, Switzerland). Primary human bronchial epithelial cells (HAE, Epithelix Sarl, Geneva, Switzerland) were expanded in BEGM in-house (Lenart et al., 2023). Calu-3 cells (HTB-55, ATCC) were grown in EMEM medium containing Minimal Essential Medium (MEM, Biowest, Nuaillé, France) supplemented with non-Essential Amino Acid (dilution 1:100) (Biowest), 1 mM sodium pyruvate (Biowest) and 10% fetal bovine serum (FBS; Biowest). Cells were maintained at 37°C under 5% CO_2_.

### 2.2 Air-liquid interface cell differentiation

Cells were differentiated to the air-liquid interference model according to a protocol described previously (Jakiela et al., 2008) with some modifications. Briefly, 2 × 10^5^ BEAS-2B cells were suspended in BEGM medium at the top of Thincert™ culture inserts (Greiner Bio-One, Kremsmünster, Austria) previously coated with 0.05 mg/mL collagen (Merck, Darmstadt, Germany). Inserts were submerged into BEGM to achieve full cell confluence. Next, the apical medium was discarded and 700 µL of differentiation medium were added to the basolateral side. Differentiation medium consisted of Dulbecco’s Modified Eagle’s Medium (DMEM, Sigma-Aldrich, St. Louis, MO, USA) and BEGM (Lonza) at proportion of 1:1 supplemented with 80 nM retinoic acid (RA). Cells were cultured for four weeks with weekly medium changing to obtain fully differentiated ALI model. In the case of Calu-3 cells, 1.35 × 10^5^ cells were used for ALI preparation. Cell differentiation was achieved after two weeks in differentiation medium (EMEM medium supplemented with 10% FBS and 80 nM RA). The HAE ALI model was performed according to a previously reported procedure (Lenart et al., 2023).

### 2.3 Microbial cultures

#### 2.3.1 Bacterial growth conditions

An invasive strain of *Porphyromonas gingivalis* W83 (ATCCBAA-308 and an isogenic triple gingipain-null mutant ΔKΔRAB (ΔrgpA, ΔrgpB, Δkgp), were used in this study (Benedyk et al., 2016). These strains were cultivated under anaerobic conditions (90% N_2_, 5% H_2_, and 5% CO_2_) at 37°C on agar plates (BTL, Lodz, Poland) with the addition of sheep blood (5% (v/v) or in liquid tryptic-soy broth (TSB) (30 g/L; Merck) supplemented with 5 g/L yeast extract (Bioshop, Burlington, Canada), L-50 μg/mL cysteine, 5 μg/mL hemin, and 0.5 μg/mL vitamin K (all from Merck). For ΔKΔRAB, additionally 1 µg/mL tetracycline (Merck) was added. After overnight liquid culture, the bacteria were centrifuged (5000×g, 30 min), washed and resuspended in phosphate-buffered saline pH 7.4 (PBS; Biowest). To estimate the number of cells in the suspension, the optical density at 660 nm was measured.

#### 2.3.2 Fungal growth conditions

The *Candida albicans* strains 3147 (ATCC 10231) and SC5314 (ATCC MYA-2876) were cultured under aerobic conditions in liquid YPD medium containing 1% yeast extract, 2% glucose, 2% soybean peptone (all components from Merck) for 18 h at 30°C and 170 rpm in an orbital rotary shaker MaxQ 6000 (Thermo Fisher Scientific, Waltham, MA, USA). Fungal cells were centrifugated (3000×g, 5 min), washed, and resuspended in PBS to estimate the number of cells in the suspension by measuring optical density at 600 nm.

#### 2.3.3 Formation of mono- (homotypic) or dual-species (heterotypic) biofilms

*C. albicans* (1 × 10^7^ CFU/mL) or *P. gingivalis* (1 × 10^8^ CFU/mL) cells were seeded in RPMI 1640 medium (Biowest) supplemented with 10% FBS for biofilm formation. Bacterial biofilm was formed under anaerobic conditions in a GENbox jar anaerobic generator (bioMérieux, Craponne, France) for 48h at 37°C. Fungal or dual-species biofilm were formed under aerobic conditions in a humidified atmosphere of 95% air and 5% CO_2_ for 48h at 37°C. Next, the supernatants were collected, centrifuged twice (3000×g, 5 min and 6000×g, 20 min) and filtered with 0.22 µm filters. Sterile supernatants were aliquoted and stored at −80°C for host cell treatment.

### 2.4 Models of infection

Two infection models were developed: one in which biofilm was formed directly on the epithelial surface and a second in which the epithelium was treated with supernatants collected from the biofilm, hereinafter referred to as BIOFILM and SUPERNATANT, respectively.

In the BIOFILM model, *C. albicans* 3147 (1×10^7^ CFU/mL) or *P. gingivalis* W83 or ΔKΔRAB (1×10^8^ CFU/mL) or both cell types at a yeast-to-bacteria ratio of 1:10 were added to the apical side of the human epithelial ALI model. Cells on the basolateral side were suspended in RPMI 1640 (Biowest) supplemented with 10% FBS. Infected epithelium was incubated for 6 or 24 hours at 37°C under aerobic conditions at 5% CO_2_. In the SUPERNATANT model, supernatants at different dilutions were added to the apical and basolateral sides of the epithelium ALI cells and incubated for 24 hours under conditions analogous to the BIOFILM model. After stimulation, the medium from the bottom of the insert was collected and stored at −80°C for cytokine measurement, while the human cells were used for further protein analyses.

### 2.5 Measurement of the mitochondrial activity of human epithelial cells in the BIOFILM model

The mitochondrial activity of differentiated BEAS-2B cells after 24-hour direct contact with biofilms was detected using MitoTracker™ Orange CMTMRos (Thermo Fisher Scientific). After washing, the cells were treated with 1 µM MitoTracker™ Orange CMTMRos dye for 30 min at 37°C. Next, cells were fixed with 4% formaldehyde (p-FA) for 20 minutes and washed with PBS. Chitin staining of yeast cells was performed with an additional treatment using 0.1 mg/mL Calcofluor White (Fluka, Buchs, Switzerland) for 10 minutes.

The membranes were detached from the insert with a scalpel and mounted onto glass slides with ProLong™ Glass Antifade Mountant (Invitrogen, Waltham, MA, USA). Fluorescence was quantified using the Leica Stellaris 5 confocal microscope (Leica Microsystems, Mannheim, Germany) equipped with 20 mW laser diodes (488 nm, 561 nm). Based on the Z-stack images obtained, the fluorescence signal for human cells in the maximum Z projection was measured. To exclude potential interference from yeast cell mitochondria in the fluorescence measurements, the MitoTracker™ Orange signal from regions defined by Calcofluor White Stain (Merck) fluorescence was subtracted. Image analyses were performed with Fiji software (ImageJ, version 1.54f (USA)). (More detailed description is included in the **Supplementary Material**).

### 2.6 Measurement of metabolic activity of human epithelial cells in the SUPERNATANT model

The metabolic activity of differentiated BEAS-2B cells was measured using the XTT assay (sodium 3′-[1-(phenylaminocarbonyl)-3,4-tetrazolium]-bis(4-methoxy-6-nitro)benzene sulfonic acid hydrate, Invitrogen). Cells were incubated for 24 hours with previously prepared 10% and 100% supernatants from biofilms, which were added to the top and bottom sides of the insert. After stimulation, cells were carefully washed with PBS and incubated with mixture containing 1 mg/mL XTT and 5 mg/mL phenazine metosulfate (Merck). Following a one-hour incubation at 37°C in the dark, the mixture was transferred to a 96-well plate and absorbance was measured at 450 nm using a PowerWaveX microplate reader (BioTek Instruments, Winooski, VT, USA).

### 2.7 Epithelial cell permeability assay

Differentiated cells in culture inserts were incubated with filtered 50% of supernatant from mono- or dual-species biofilm for 24 hours. After this time, the supernatant was removed and replaced in the bottom side of inserts with a fresh aliquot of phenol red-free RPMI 1640 medium. Epithelial cell permeability was assessed by adding a solution of 70 µg/mL dextran (70 kDa) conjugated with fluorescein isothiocyanate (FITC) (Merck) to the apical side of the cells. After 3 hours incubation, the fluorescence of the medium collected from the bottom side was measured at λ_ex_= 485 nm and λ_em_=535 nm using the Synergy H1 microplate reader (Biotek, Winooski, VT, USA).

### 2.8 Penetration of the epithelium by *C. albicans*

The progression of epithelium penetration by yeast cells was analyzed with three biofilm variants formed in direct contact with the surface of host cells in the ALI model: i) *C. albicans* SC5314, ii) mixed biofilm with a yeast-to-*P. gingivalis* W83 ratio of 1:10, and iii) *C. albicans* SC5314 with supernatant derived from an anaerobic culture of *P. gingivalis* W83. All biofilms were formed in phenol red-free RPMI 1640 during 24 hours at 37°C under aerobic conditions. Next, the inserts were fixed with 4% p-FA for 20 minutes, and yeast cell localization was analyzed using specific staining protocols. Briefly, cells were permeabilized for 10 minutes with 0.5% Triton X-100. F-actin in human cells was stained with 40-fold diluted phalloidin solution labelled with Alexa Fluor™ 568 (ThermoFisher Scientific) for 1 h at room temperature. Subsequently, mannans in the yeast cell walls were stained by incubating the membranes with 100 µg/mL concanavalin A (conA) conjugated with Alexa Fluor™ 488 (Thermo Fisher Scientific) for 30 minutes at room temperature. The membranes containing cells were detached from the insert using a scalpel. Samples were mounted with ProLong™ Glass Antifade Mountant (Invitrogen) and imaged using a Leica Stellaris 5 confocal microscope. Quantitative assessment of *C. albicans* penetration into epithelial layers was performed by generating a fluorescence profile along the Z-axis for each channel, illustrating the relationship between fluorescence intensity and sample depth. The confocal Z-stacks were analyzed using Leica LasX software (Leica Microsystems, Mannheim, Germany). The fluorescence intensity was measured across the depth of the sample, and the distance between peak mean fluorescence values was calculated, enabling direct comparisons of the extent of invasion under different experimental conditions.

### 2.9 Immunofluorescence staining of proteins

HAE and Calu-3 cells were treated for 24 hours with sterile 50% of mixed biofilm supernatant collected from 48-hours culture of *C. albicans* 3147 and *P. gingivalis* W83 culture. Cells were fixed with 4% p-FA at room temperature (RT) for 10 minutes. Next, the cell membrane was permeabilized by adding 0.1% Triton X-100 for 15 minutes at the same temperature. After washing with PBS, the cells were blocked with 2% BSA (BioShop Canada Inc., Burlington, Canada) for 2 hours at RT. An appropriate dilution of primary antibody in 1% BSA was then added as shown in **Table 1** and samples were incubated overnight at 4°C. The following day, cells were washed with PBS, and the required concentration of secondary antibody in 1% BSA (**Table 1**) was added for 1 hour at RT. Cell nuclei were stained with 5 ug/mL of Hoechst 33342 for 10 min at RT. After the staining procedure, membranes were excised from the insert with a scalpel and mounted with ProLong™ Glass Antifade Mountant (Invitrogen). Images were captured with Leica Stellaris 5 confocal microscope.

**Table 1:** List of antibodies used for immunofluorescence staining.

Primary antibodies	Host	Dilution	Company	Secondary antibodies	Host	Dilution	Company
ZO-1	Rabbit	1:100	Invitrogen	Anti-rabbit Alexa Fluor 488	Donkey	1:1000	Abcam
β-tubulin	Mouse	1:50	Abcam, Cambridge, UK	Anti-mouse Alexa Fluor 647	Donkey	1:1000	Abcam
MUC5AC	Mouse	1:100	Merck	Anti-mouse Alexa Fluor 647	Donkey	1:1000	Abcam

### 2.10 Western blot analysis

BEAS-2B cells in the SUPERNATANT infection model were incubated with sterile 50% supernatants for 24 hours at 37°C under aerobic conditions. After incubation, harvested cells were resuspended in RIPA buffer (Merck) supplemented with protease inhibitor cocktail (Promega, Mannheim, Germany), 1 mM EGTA, 5 mM EDTA, and gingipain inhibitors (1 μg/mL KYT-1, 1 μg/mL KYT-36). Total protein concentration in the lysates was determined using the Pierce BCA Protein Assay Kit (ThermoFisher Scientific) according to the manufacturer’s instructions. Samples containing 8 μg of protein were resolved in 8% or 12% SDS-PAGE, and protein detection was performed following the procedure described previously (Fandiño-Devia et al., 2024). The primary and secondary antibodies used in this study are listed in **Table 2**. Protein levels were quantified by densitometric analysis of the bands using QuantityOne 1-D Analysis Software (Bio-Rad Laboratories, USA) with GAPDH used as a loading control.

**Table 2:** List of antibodies used for Western blot analysis.

Primary antibodies	Host	Dilution	Company	Secondary antibodies	Host	Dilution	Company
ZO-1	Rabbit	1:1000	Invitrogen	Anti-rabbit	Goat	1:1000	R&D Systems, Minnesota, US
E-cadherin	Mouse	1:1000	BD Biosciences, NY, US	Anti-mouse	Goat	1:1000	R&D Systems
β-tubulin	Mouse	1:500	Abcam	Anti-mouse	Goat	1:10000	R&D Systems
HO-1	Rabbit	1:1000	Cell Signaling Technology, Danvers, Massachusetts, USA	Anti-rabbit	Goat	1:5000	R&D Systems
Bax	Rabbit	1:1000	Cell Signaling Technology	Anti-rabbit	Goat	1:1000	R&D Systems
BcL-xL	Rabbit	1:2000	Cell Signaling Technology	Anti-rabbit	Goat	1:1000	R&D Systems
GAPDH	Mouse	1:3000	Cell Signaling Technology	Anti-mouse	Goat	1:10000	R&D Systems

### 2.11 IL-8 measurement

Levels of IL-8 produced by the human epithelium were quantified in medium collected from the bottom part of the insert of the two infection models using the Human IL-8 ELISA Set (BD Biosciences, San Diego, CA, USA OptEIA™) following the manufacturer’s instructions.

### 2.12 Evaluation of *in vivo* toxicity of *C. albicans* and *P. gingivalis* biofilm using *Galleria mellonella* model

Dual-species biofilm of *C. albicans* and *P. gingivalis* W83 were formed as described above. A mixture of amphotericin B (at final concentration of 0.5 or 1 µg/mL) and levofloxacin (at final concentration of 1 or 10 µg/mL) was added to 24-hour formed biofilm and incubated for an additional 24 hours. Following this period, the supernatants were collected and filtered with 0.22 µm Ultrafree-CL Centrifugal Filters (Merck) to sterilize the samples. The potential toxicity of *C. albicans* and *P. gingivalis* mixed-species biofilm was evaluated in *Galleria mellonella* larvae according to an established protocol (Kulig et al., 2024). Groups of 10 larvae were randomly selected and injected into the last left proleg with 10 µL of supernatants using a 10 μL Hamilton syringe (Merck). Control larvae received injections of RPMI 1640 (Biowest) alone. The larvae were incubated at 37°C, and their mortality was assessed daily by the lack of movement in response to touch. Two independent biological replicates were performed, and the average results are presented. Survival data were analyzed using the Mantel-Cox log-rank test in GraphPad Prism 7.0.

### 2.13 Statistical analysis

The data were presented as means ± SD or as means ± SEM. For statistical analysis, *t*-test or one-way ANOVA test with Dunnett’s multiple comparisons was used with GraphPad Prism software version 5.03 or 7.0 (GraphPad Software, La Jolla, CA, USA). Statistical significance levels were marked * for p < 0.05, ** for p < 0.01, *** for p < 0.001.

## 3 Results

### 3.1 Impact of microbial biofilm and its supernatants on the metabolic activity of epithelial cells

Lung epithelial cells constitute a protective barrier against the external environment. During respiration, these cells are exposed to various toxic agents, including numerous pathogens. Our study aimed to investigate the impact of two common pathogens, *C. albicans* and *P. gingivalis*, on the disruption of the epithelial barrier. The first step involved evaluating the metabolic activity of epithelial cells in direct contact with the biofilm of each pathogen, or with a mixed biofilm. For this purpose, the widely recommended ALI model was utilized, in which differentiated epithelial cells form a structure that closely mimics their physiological environment, surpassing the limitations of traditional 2D models (Silva et al., 2023). These 3D models replicate the polarized architecture of epithelial tissues and incorporate extracellular matrix (ECM) components, enhancing physiological relevance. Additionally, 3D models enable detailed analysis of tight junction integrity and barrier disruption caused by pathogens, accurately reflecting changes in epithelial permeability due to infection or inflammation. Moreover, 3D models effectively capture the complex dynamics of biofilm formation and pathogenic synergy.

In our study we use three epithelial cell lines: HAE cells, being primary cells, typically exhibit the most robust barrier function, followed by Calu-3 and then BEAS-2B. This hierarchy influences their susceptibility to bacterial invasion and the extent of structural disruption upon infection. For instance, HAE cells are ideal for studying mucociliary clearance, while Calu-3 cells are valuable for research on barrier function in the context of infection.

**Figure 1:**
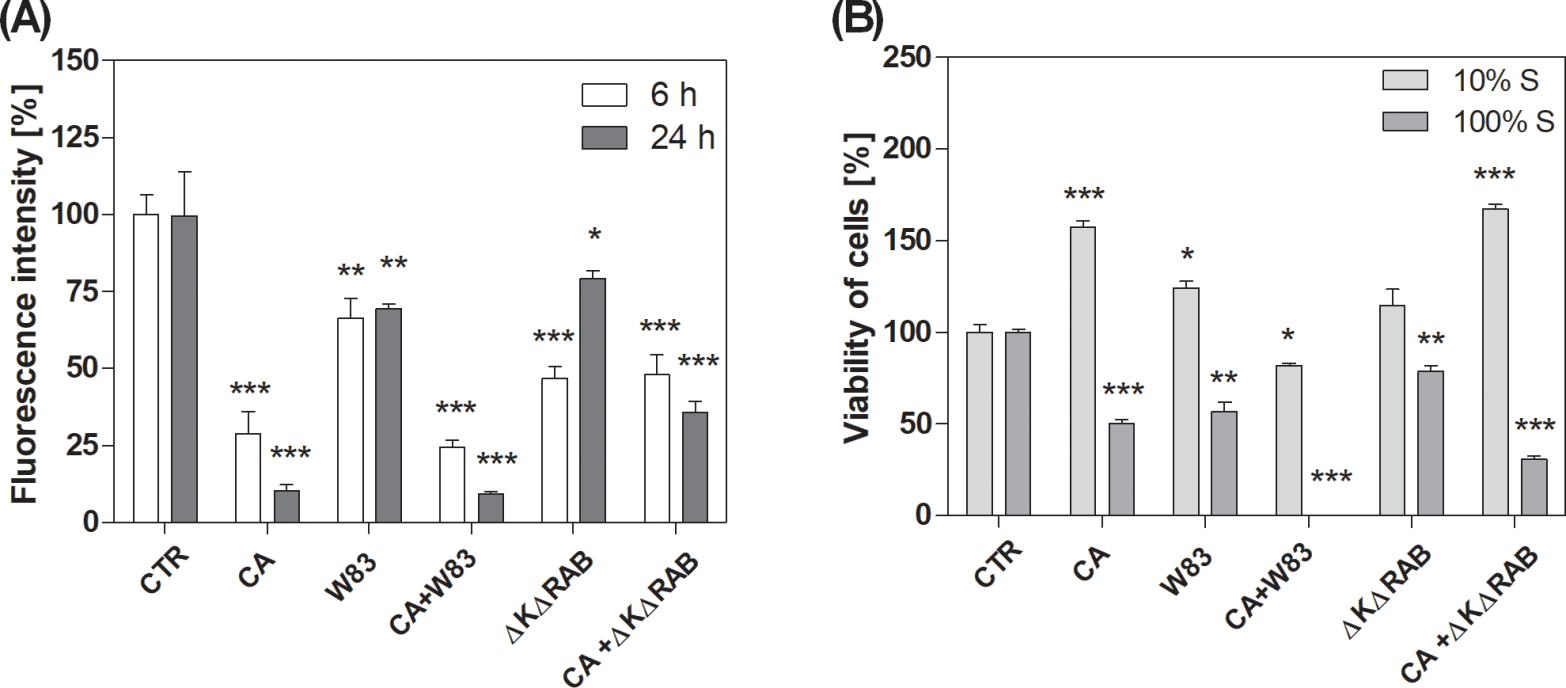
Level of mitochondrial activity of BEAS-2B cells after treatment with *C*. *albicans* and/or *P. gingivalis* biofilm **(A)** or cell viability treated with biofilm supernatants **(B)**. Mono- or dual-species biofilms were grown on BEAS-2B cells for 6 or 24 hours. Afterward, samples were stained with MitoTracker^TM^ Orange CMTMRos **(A)** and imaged using a Leica Stellaris confocal microscope. The fluorescence intensity of the epithelium was quantified using Fiji software (ImageJ). Cell viability was determined using the XTT assay **(B)**, performed after a 24-hour incubation of BEAS-2B cells with 10% or 100% sterile supernatants collected from a 48-hour biofilms culture. The graphs show the average results of three repetitions. Data are presented as mean ± SEM. An unpaired t-test was used for statistical analysis (* for p < 0.05, ** for p < 0.01, *** for p < 0.001 vs. untreated cells (CTR)). (CTR- untreated cells; CA – *C*. *albicans* 3147; W83 – invasive strain of *P. gingivalis*; ΔKΔRAB - gingipain-null mutant of *P. gingivalis*).

Mitochondrial activity in live BEAS-2B cells after direct biofilm exposure was assessed using MitoTracker™ Orange CMTMRos, a dye that selectively accumulates in active mitochondria, reflecting their metabolic function. Background fluorescence signals originating from pathogens were subtracted as described in the Materials and Methods section and **Supplementary Material**. The resulting fluorescence intensity from live cells was quantified and expressed as a percentage relative to the signal observed in untreated cells. The biofilm formed by *C. albicans* strain 3147 (CA) significantly reduced mitochondrial activity in a time-dependent manner. In contrast, the *P. gingivalis* W83 strain biofilm (W83) induced only a moderate reduction in activity, irrespective of the duration of biofilm formation (**Figure 1A**). Interestingly, the effect of mixed biofilm (CA+W83) was similar to that observed for CA biofilm alone. Epithelial cells infected with the less virulent of *P. gingivalis* strain (ΔKΔRAB) exhibited reduced cell viability; however, when exposed to mixed biofilm (CA+ΔKΔRAB), the observed effect was less pronounced than with CA+W83 biofilm. Furthermore, the ΔKΔRAB strain appeared to exert a weaker effect on epithelial cells viability after 24 hours when acting alone. Notably, in the presence of *C. albicans*, the reduction in cell viability after 24 hours became more pronounced. Importantly, the lowest viability of differentiated epithelial cells was observed in response to the mixed CA+W83 biofilm.

To investigate the contribution of active substances secreted by pathogens to the viability of epithelial cells, additional experiments were conducted. Instead of directly infecting the cells, supernatants derived from 48-hour growing biofilms were applied. These supernatants were tested at two concentrations: diluted (10%) and undiluted (100%). A significant difference in cell viability was observed depending on the concentration of the supernatant (**Figure 1B**). At 10% concentration, supernatants generally led to an increase in metabolic activity, although a downward trend was noted for cells treated with mixed CA+W83 supernatant. In contrast, the supernatant from the CA+ΔKΔRAB biofilm caused a marked increase in metabolic activity, surpassing that of other treatments. It is important to note that the observed increase in metabolic activity at 10% supernatant concentration may not directly correlate with increased cell viability. Instead, this effect may result from adaptive metabolic and mitochondrial reprogramming in response to stress-induced factors, as previously described by Stepanenko and Dmitrenko (2015). Therefore, it should not be assumed that cell viability increases in response to 10% supernatants, rather, the metabolic activity of the cells is elevated. On the other hand, the application of undiluted (100%) supernatants consistently resulted in a decrease in metabolic activity compared to untreated cells. Supernatants derived from CA and W83 biofilms significantly reduced cell metabolism, with the most pronounced reduction observed in cells treated with the mixed CA+W83 supernatant. These findings suggest that epithelial cell metabolism is disrupted by pathogens through the secretion of active substances.

### 3.2 Supernatants of mixed biofilm induce disruption of ALI cell model integrity

**Figure 2:**
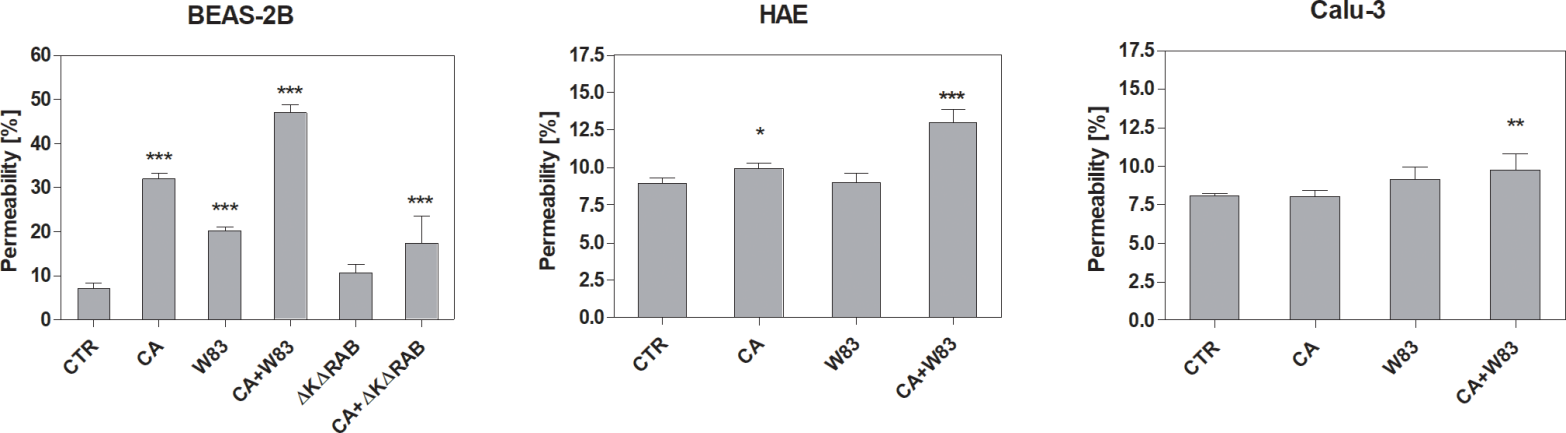
Influence of biofilm supernatants on increased epithelial cell permeability. BEAS-2B, HAE, and Calu-3 cells were cultured for 24 hours with sterile supernatants collected from single- or dual-species biofilms. After incubation, 70 kDa dextran-FITC was added to the apical side of the epithelium. The fluorescence signal, corresponding to the movement of dextran-FITC across the epithelium, was measured in the basolateral compartment after 3 hours of incubation. The graphs show the epithelial permeability relative to an insert without cells, which was defined as having 100% permeability to dextran. The data presented in the graph represents the average results of four repetitions. Data were shown as means ± SD. Statistical analysis was carried out using a one-way ANOVA with Dunnett's multiple comparisons. *for p < 0.05, ** for p < 0.01, and *** for p < 0.001 vs. untreated cells (CTR). (CTR- cells not treated with supernatant; CA – *C. albicans* 3147; W83 – invasive strain of *P. gingivalis*; ΔKΔRAB- gingipain-null mutant of *P. gingivalis*).

The permeability of the ALI cell model using various epithelial cell types was investigated after 24-hour incubation with supernatants (**Figure 2**). As expected, cells treated with supernatants derived from the mixed CA+W83 biofilm exhibited the highest permeability. BEAS-2B cells exposed to CA and W83 supernatants also demonstrated increased permeability whereas supernatants from ΔKΔRAB and CA+ΔKΔRAB biofilms were less effective in inducing this effect. In experiments involving other cell types (HAE and Calu-3), supernatants from ΔKΔRAB and CA+ΔKΔRAB biofilms were excluded from the analysis. Nonetheless, a significant increase in permeability to FITC-dextran was observed in HAE and Calu-3 cells following treatment with supernatants from the mixed CA+W83 biofilm.

### 3.3 *P. gingivalis* enhances *C. albicans* penetration in host epithelial cells

**Figure 3:**
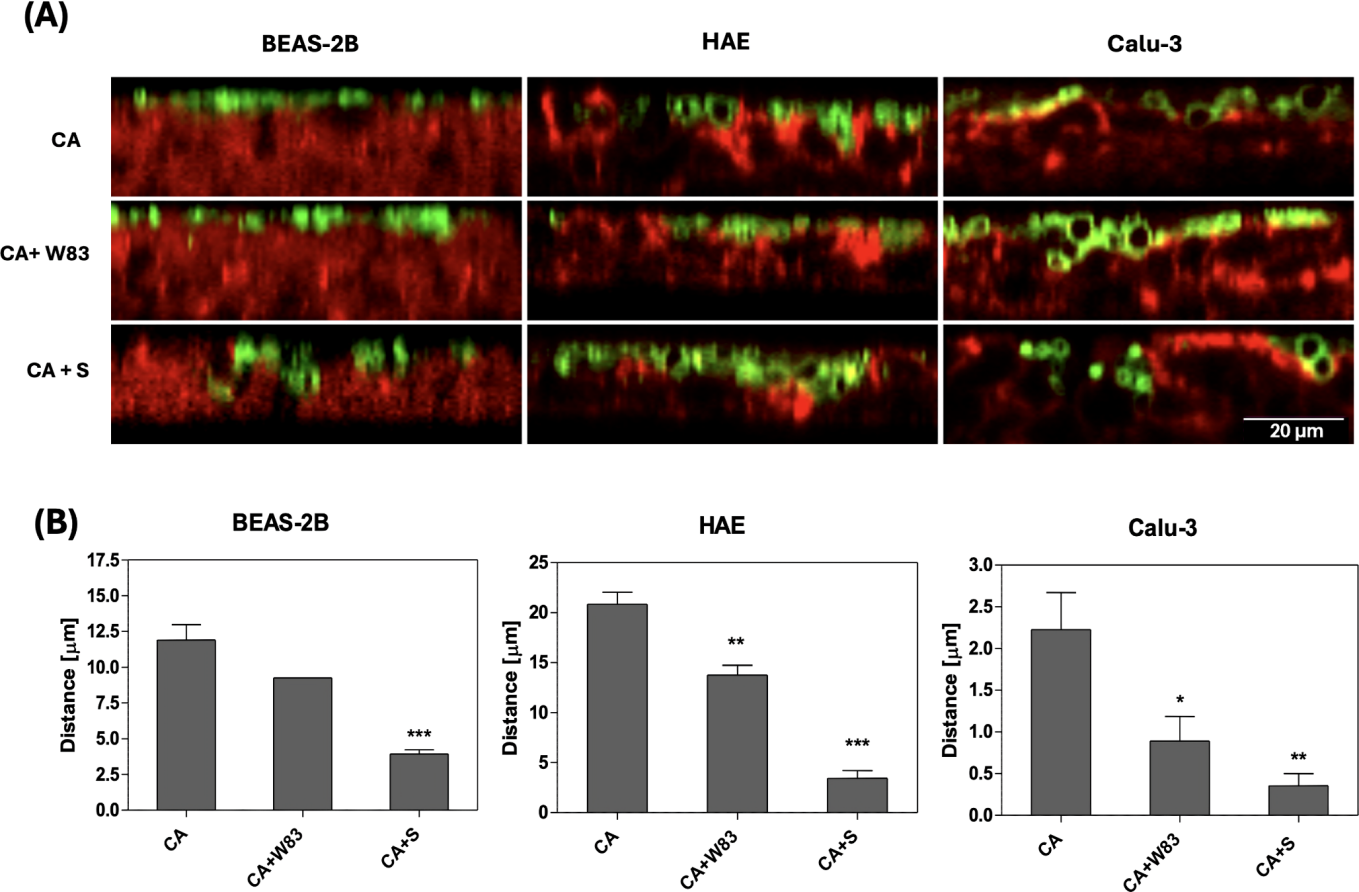
Effect of *P. gingivalis* on the progression of airway epithelium invasion by *C. albicans* biofilm. BEAS-2B, HAE, Calu-3 cells were cultured for 3 hours in the presence of *C*. *albicans* SC5314; a mixed biofilm of *C*. *albicans* SC5314 and *P. gingivalis* W83; or *C*. *albicans* SC5314 with 50% supernatant from a 48-hour anaerobic culture of *P. gingivalis* W83. *C*. *albicans* was labelled with 100ug/mL ConA-FITC, while F-actin in human cells was stained with phalloidin conjugated to Alexa Fluore568 (dilution 1:40). Representative orthogonal view images obtained using a Leica Stellaris 5 confocal microscope illustrate the penetration progress of *C*. *albicans* (green) inside the epithelium (red), in the presence of *P. gingivalis* or bacterial supernatant **(A)**. The graphs display the position of the yeast biofilm relative to the human cells **(B)**, determined using the stack profile plot function in Leica Las X software. The data show the results of the quantitative analysis from three randomly selected locations within the sample. Data are presented as means ± SD. The statistical analysis was performed using a one-way ANOVA with Dunnett's multiple comparison. * for p < 0.05, ** for p < 0.01, and *** for p < 0.001 vs. CA. (CA – *C*. *albicans* SC5314; W83 – invasive strain of *P. gingivalis*; S – 50% of supernatant collected from *P. gingivalis* W83 anaerobic culture).

Since supernatants from pathogen biofilms were shown to induce degradation of the epithelial 3D cell model, further studies were conducted to determine whether *C. albicans* cells can penetrate this barrier. Differentiated ALI epithelial cells were infected with yeast cells alone (CA), a mixture of *C. albicans* and *P. gingivalis* (CA+W83), or a mixture of *C. albicans* and supernatant derived from an anaerobic culture of *P. gingivalis* (CA+S). The localization of the maximal number of *C. albicans* cells in the epithelial layer was quantified using confocal microscopy as described in the Materials & Methods section. Representative images of *C. albicans* (green) penetration on epithelial cells (red) as orthogonal projections of Z-stack are shown in **Figure 3A**. The distance between the basolateral side of the epithelial layer and the location of *C. albican*s cells was significantly shorter when yeast cells were co-cultured with *P. gingivalis*, suggesting cooperative interactions between the two pathogens that facilitates *C. albicans* penetration (**Figure 3B**). Moreover, infection with yeast in the presence of supernatant from an anaerobic *P. gingivalis* biofilm (CA+S) further enhanced *C. albicans* penetration, supporting our earlier hypothesis regarding the role of secreted factors in promoting barrier disruption. All tested 3D cell models, including HAE and Calu-3, exhibited a similar trend, with increased susceptibility to *C. albicans* penetration in the presence of *P. gingivalis* or its supernatants. These results highlight the synergistic effects of these pathogens in compromising epithelial barrier integrity.

### 3.4 Impact of *C. albicans* and *P. gingivalis* on epithelial barriers

**Figure 4:**
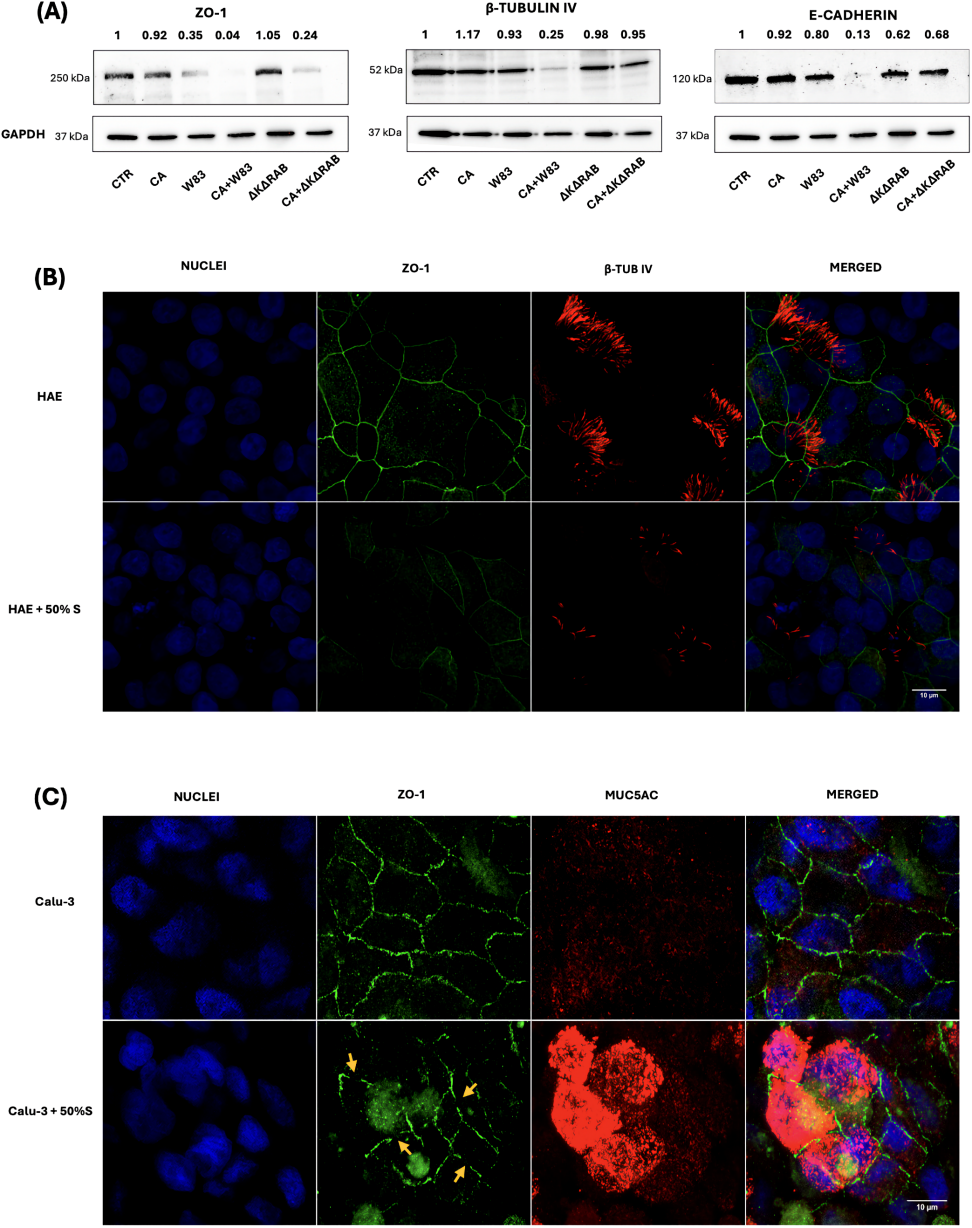
Changes in the level of epithelial barrier proteins influenced by the supernatant of single- or dual-species biofilm. BEAS-2B cells were incubated with 50% biofilm supernatants for 24 hours. Protein expression levels of ZO-1, β-tubulin IV and E-cadherin were analyzed by western blotting **(A)** with detection using chemiluminescent substrate. GAPDH was used as an internal control for protein loading. In the representative image, changes in protein expression were quantified relative to the signal of the control sample (CTR), for which the signal level was defined as 1. The panels below illustrate the change in levels of β-tubulin IV (red) in HAE cells **(B)** or MUC5AC in Calu-3 cells **(C)** and the decrease in ZO-1 protein levels (green) in both cell types, after 24-hours treatment with 50% of biofilm supernatants. The orange arrows indicate the loss of ZO-1 protein during tight junction formation in Calu-3 cells. Samples were analyzed microscopically (Leica Stellaris 5) and the obtained images were presented as a maximum Z projection. Panel A shows a representative result from three biological replicates, while Panel B and C illustrate an image representative of three randomly selected sites within the sample. (CTR- BEAS-2B cells non treated with supernatant; CA - C*. albicans* 3147; W83 – invasive strain of *P. gingivalis*; ΔKΔRAB- gingipain-null mutant of *P. gingivalis;* S – supernatant from *P. gingivalis* W83 biofilm, cultured in anaerobic conditions).

Tight junctions (TJs) are intercellular adhesion complexes that form a paracellular diffusion barrier, regulating epithelial permeability (Furuse and Takai, 2021). Numerous proteins are involved in TJs, and their degradation by proteases secreted during infection has been linked to increased epithelial permeability. Considering our findings of increased epithelial permeability and *C. albicans* penetration, the next step was to examine the status of TJ and adhesion associated proteins, such as ZO-1 or E-cadherin. In the ALI BEAS-2B model, ZO-1 and E-cadherin levels were analyzed by western blotting (**Figure 4A**). The supernatants from W83 biofilm caused a reduction in ZO-1 expression, while the CA+W83 supernatant led to an almost complete loss of the protein. No significant changes in ZO-1 expression were observed in cells treated with supernatant derived from CA or ΔKΔRAB. However, a marked reduction in ZO-1 levels was detected following treatment with CA+ΔKΔRAB supernatant. Similar results were observed for E-cadherin, where cells exposed to CA+W83 supernatant showed a strong reduction in protein levels. Interestingly, unlike ZO-1, E-cadherin levels were only slightly reduced following treatment with CA+ΔKΔRAB supernatant.

Other critical elements involved in capturing and reversing the trajectory of inhaled pathogens are mucus and cilia lining the airways. In BEAS-2B cells, the presence of cilia was assessed by detecting β-tubulin IV (β-TUB IV). Results indicated that only supernatant CA+W83 caused a significant reduction in β-TUB IV levels. Given that active substances secreted by *P. gingivalis* W83 appear to promote increased epithelial permeability and facilitate *C. albicans* penetration, further studies were conducted using HAE and Calu-3 cells to check changes in ZO-1 and β-TUB IV levels after treatment with supernatant from biofilm collected from 48- hours anaerobic culture of W83. In the ALI HAE cell model, immunofluorescence analysis revealed decreased ZO-1 levels and reduced β-TUB IV detection following supernatant treatment (**Figure 4B**). In 3D Calu-3 cell model, the level of the MUC5AC mucin, a key glycoprotein in airway mucus with a critical role in pathogen defense (Dhanisha et al., 2018), was visualized with immunofluorescence. Overexpression of MUC5AC was observed in Calu-3 cells treated with CA+W83 supernatant (**Figure 4C**). Concurrently, a disturbance in ZO-1 expression was noted, suggesting protein degradation.

### 3.5 Epithelium cell response to *C. albicans* and *P. gingivalis* biofilms

**Figure 5:**
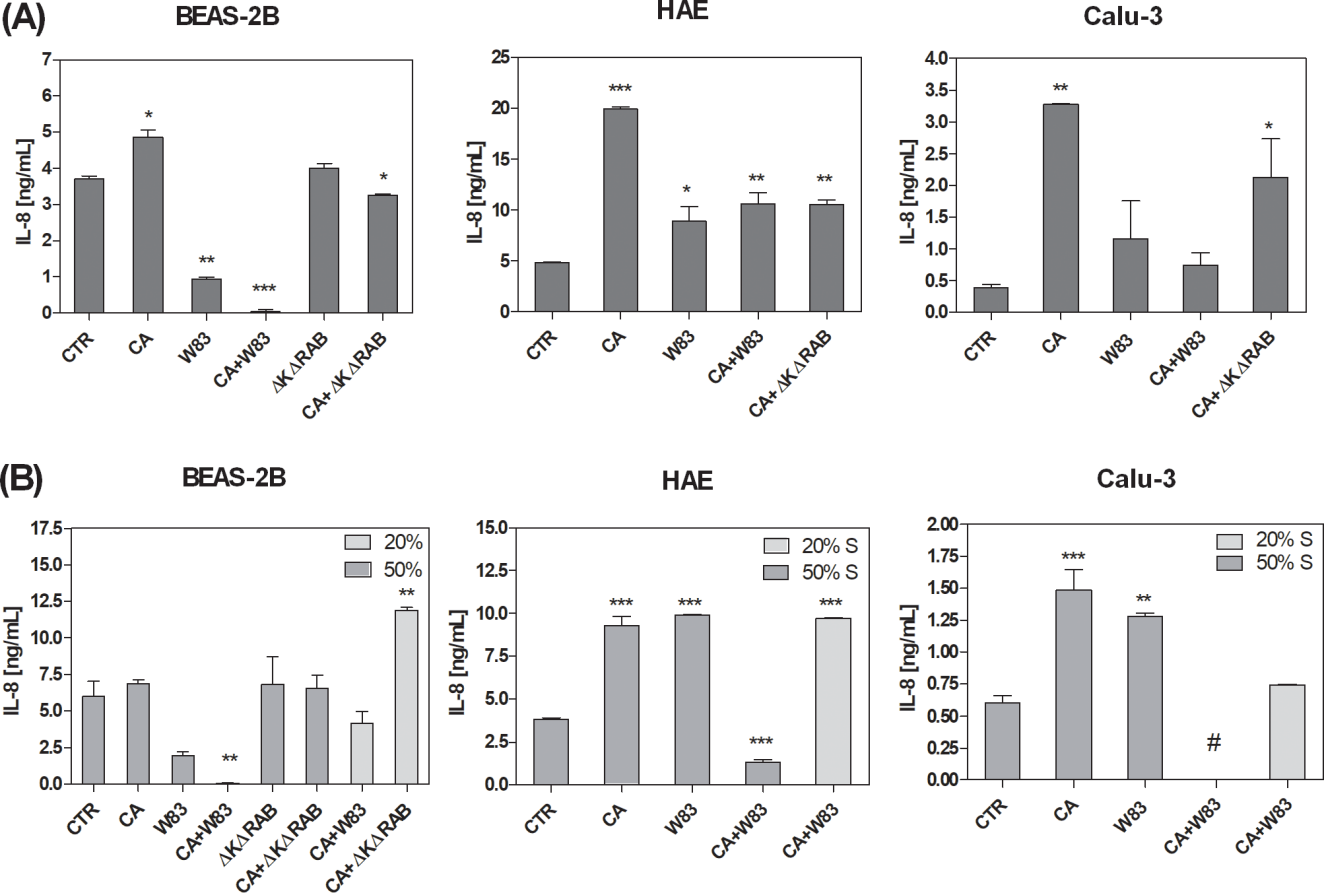
Alterations in IL-8 secretion by the airway epithelium as a consequence of exposure to *C. albicans* or/and *P. gingivalis* biofilm **(A)** or supernatants **(B)**. BEAS-2B, HAE and Calu-3 cells were in contact for 24 hours with homotypic or heterotypic biofilm **(A)** or with 20% or 50% supernatants from aerobic biofilm culture **(B)**. IL-8 concentrations were quantified in medium collected from the basolateral side of the cells using a dedicated ELISA kit (BD Biosciences). The graphs show the average results from three independent repetitions. The data are presented as means ± SD. Statistical analysis was performed using one-way ANOVA with Dunnett's multiple comparison. * for p < 0.05, ** for p < 0.01, and *** for p < 0.001 vs. untreated cells (CTR). (CTR – cells non treated with biofilm or supernatant, CA – *C. albicans* 3147; W83 – invasive strain of *P. gingivalis*; S – supernatant collected from biofilm; # - undetected).

The disruption of TJs and the consequent loss of epithelial barrier function enable the penetration of harmful agents, triggering mechanisms that contribute to the development of chronic inflammation (Ogulur et al., 2024). In this context, interleukin 8 (IL-8), a cytokine with pivotal roles in pro-inflammatory responses (Matsushima et al., 2022), was analyzed to elucidate the impact of *C. albicans* and *P. gingivalis* on the propagation of inflammatory processes in epithelial cells. The investigation aimed to elucidate how these pathogens influence IL-8 secretion, thereby modulating the inflammatory milieu and potentially exacerbating epithelial damage. ALI cell models were exposed to mono- or dual-species biofilms formed with CA, W83, or ΔKΔRAB. IL-8 concentrations were measured 24 hours post-infection (**Figure 5A**). The CA biofilm induced the highest level of cytokine secretion in all cell types. However, a heterotypic biofilm formed with the W83 strain attenuated the IL-8 secretion triggered by CA. Similarly, a heterotypic CA+ΔKΔRAB biofilm also reduced IL-8 levels, although the decrease observed in BEAS-2B and Calu-3 cells was moderate. Notably, the W83 biofilm alone did not significantly increase IL-8 production, which could be attributed to the fact that in these experimental conditions, the *P. gingivalis* biofilm lacked strict anaerobic conditions, potentially affecting its virulence.

Changes in IL-8 secretion by epithelial cells were analyzed following exposure to biofilm supernatants. The ALI BEAS-2D cell model showed significantly lower IL-8 levels after treatment with W83 supernatant (**Figure 5B**). Furthermore, the mixed supernatant of *C. albicans* and *P. gingivalis* W83 (CA+W83) resulted in a substantial reduction in IL-8 concentration, particularly when a less diluted supernatant (50%) was used. In contrast, treatment with a more diluted supernatant (20%) led to a moderate decrease in cytokine levels. Interestingly, supernatants derived from ΔKΔRAB and CA+ΔKΔRAB biofilms caused no change in IL-8 levels at higher concentrations (50%), while an increase in cytokine secretion was observed with 20% diluted supernatant. Similar concentration-dependent effects were observed in other 3D cell models. In both HAE and Calu-3 cells, IL-8 levels were significantly reduced after incubation with a 50% diluted mixed biofilm supernatant, whereas greater dilution led to higher cytokine concentrations. In contrast, supernatants derived from homotypic biofilms caused a significant increase in IL-8 concentration across all tested cell models.

**Figure 6:**
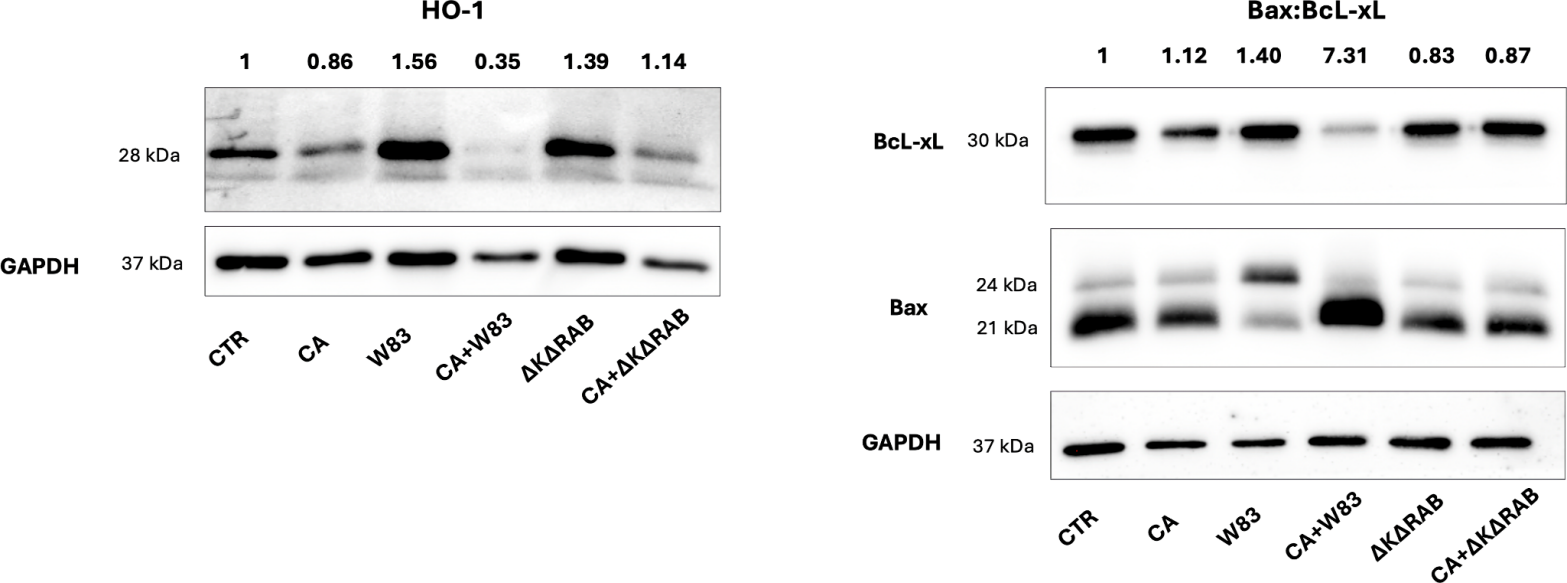
Analysis of HO-1 and Bax/BcL-xL protein expression after stimulation with supernatants from *C. albicans* or/and *P. gingivalis* biofilms. BEAS-2B cells were stimulated for 24 hours with sterile 50% supernatants from the biofilm, and then the expression levels of HO-1, Bax and BcL-xL proteins were analyzed by Western blotting. GAPDH was used as an internal control for protein loading. Changes in protein expression were calculated by densitometric analysis of the bands relative to the control sample signal (CTR), which was set to 1. The data show a representative result from three biological replicates. (CTR- BEAS-2B cells non treated with supernatant; CA- *C. albicans* 3147; W83 – invasive strain of *P. gingivalis*; ΔKΔRAB- gingipain-null mutant of *P. gingivalis;* S – supernatant from heterotypic biofilm).

Oxidative stress is widely recognized as being closely associated with the disruption of TJs and the dysfunction of the epithelial barriers (Rao, 2008). The stress-response protein heme oxygenase-1 (HO-1) was investigated to assess the effects of *C. albicans* and aggressive *P. gingivalis* strain W83 on oxidative stress in epithelial cells. HO-1 plays a critical role in the regulation of cell proliferation, differentiation and apoptosis, exerting protective effects against oxidative injury, modulating inflammation and regulating apoptotic pathways (Loboda et al., 2016). Treatment with *P. gingivalis* W83 supernatant caused HO-1 overexpression of HO-1, whereas exposure to the mixed supernatant (CA+W83) resulted in a marked reduction in HO-1 levels in the 3D BEAS-2B cell model (**Figure 6**). Similar reductions in HO-1 expression were observed with supernatants from ΔKΔRAB and CA+ΔKΔRAB biofilms. Conversely, CA supernatant alone caused a slight decrease in HO-1 levels. A quantitative analysis of HO-1 expression relative to the control GADPH protein is shown at the top of the image, with untreated cells set as the baseline (value = 1). Given that uncontrolled production of reactive oxygen species (ROS), coupled with insufficient neutralization, can trigger apoptosis, further experiments were conducted to examine the expression of proteins from the Bcl-2 family, which are involved in apoptosis regulation.

The levels of the anti-apoptotic protein Bcl-xL and pro-apoptotic proteins Bax α (21 kDa) and Bax β (24 kDa) were evaluated in 3D BEAS-2B cells following treatment with the supernatants (**Figure 6**). The CA and CA+W83 supernatants reduced Bcl-xL expression; however, only the CA+W83 supernatant induced a significant increase in Bax protein levels, measured as total expression of Bax α and Bax β. Quantitative analysis of the Bax/Bcl-xL ratio revealed strong pro-apoptotic activity for the CA+W83 supernatant, showing a seven-fold increase relative to untreated cells. In this case, the predominant form was α-Bax. In contrast, the W83 supernatant alone produced a moderate pro-apoptotic effect and, uniquely, caused an increase in the Bax β isoform. Both Bax α and Bax β are ubiquitously expressed in human cells, but Bax β is upregulated under apoptotic stimuli through inhibition of its ubiquitination (Fu et al., 2009). These findings suggest distinct mechanisms of action for *P. gingivalis* W83 when acting alone versus in combination with *C. albicans*. Notably, no significant changes in apoptotic markers were observed when epithelial cells were treated with P*. gingivalis* ΔKΔRAB supernatant, either alone or in combination with *C. albicans*.

### 3.6 The presence of *P. gingivalis* in the mixed biofilm reduces survival of *Galeria mellonella* larvae

**Figure 7:**
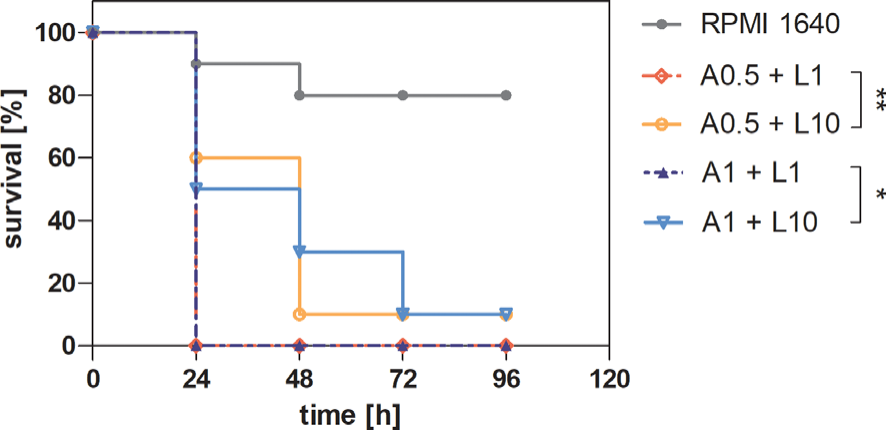
Survival curve of *Galleria mellonella* larvae after injection of supernatants from a mixed-species biofilm of *C. albicans* and *P. gingivalis*, previously treated with antibiotic and antimycotic therapy. A 24-hour biofilm of *C. albicans* 3147 and *P. gingivalis* W83 was treated with 0.5 or 1 μg/mL amphotericin B and 1 or 10 μg/mL levofloxacin for additional 24 hours. Supernatants derived from the treated biofilms were then collected and injected into *G. mellonella* larvae, with larvae injected with RPMI 1640 serving as a control. Larval survival was monitored over 96 hours. The representative results of two independent experiments are presented. Data were analyzed using the Mantel-Cox log-rank test with GraphPad Prism 7.0 software, and the results were statistically significant (p < 0.0001. Comparing the selected groups statistical significance was determined at p-value < 0.05 (* for < 0.05 and ** < 0.01). ( A(0.5 or 1)+ L(1 or 10) - supernatant from biofilm after treatment 0.5 or 1 μg/mL amphotericin B and 1 or 10 μg/mL levofloxacin).

*Galleria mellonella* larvae, a widely accepted infection model (Menard et al., 2021; Ottaviano et al., 2021; Kulig et al., 2024), were used to assess viability following treatment with supernatants derived from *C. albicans* and *P. gingivalis* biofilms in the presence of antibacterial (levofloxacin) or antimycotic (amphotericin B) drugs. Supernatants collected from biofilms treated with 0.5 μg/mL or 1μg/mL amphotericin and 1 or 10 μg/mL levofloxacin were injected into *G. mellonella* larvae, and viability was monitored over 4 days (**Figure 7**). Larvae injected with supernatants from heterotypic biofilms treated with 1 μg/mL levofloxacin exhibited the shortest survival times, regardless of the amphotericin B concentration. This indicates that lower antibiotic doses are insufficient to counteract the pathogenic effects of dual-species biofilms. Treatment with 10 μg/mL levofloxacin significantly extended larval survival, suggesting a dose-dependent effect. The most pronounced reduction in survival was observed in larvae injected with supernatants from untreated mixed-species biofilms, reinforcing the hypothesis of cooperative virulence between the two pathogens. Improved larval survival at higher levofloxacin concentrations likely correlates with reduced virulence factor production, such as gingipains, by *P. gingivalis* W83. These results highlight the complex interplay between biofilm composition, antimicrobial treatment, and pathogen virulence. Dual-species biofilms demonstrate greater resilience and induce higher pathogenicity compared to single-species biofilms. The data suggest that dual-species biofilms are more resilient and induce higher pathogenicity compared to single-species biofilms. Effective antimicrobial strategies, particularly higher doses of antibiotics like levofloxacin, are crucial in reducing the virulence of mixed biofilms, highlighting the importance of dose optimization in polymicrobial infections.

## 4 Discussion

The formation of mixed biofilms, regardless of infection type, represents a typical state of microbial coexistence. However, the relationships between microorganisms within these biofilms are dynamic, depending on the immediate needs of the microbial consortium or the dominance of a specific pathogen.

*C. albicans* is a unique microorganism capable of adapting to various environmental conditions and coexisting with different microbial species. It also demonstrates plasticity in host environments, functioning as both a commensal and a pathogen. In this study, focusing on aspiration pneumonia, we examined the interaction between *C. albicans* and *P. gingivalis* - the model which reflects the particular infectious capabilities of these organisms in individuals with impaired immunity, favoring *C. albicans*, or those with advanced periodontal disease, often dominated by *P. gingivalis*. While their coexistence in mixed infections has been documented, the exact interactions between these pathogens, particularly in the context of host epithelial cells, remain poorly understood. Our previous studies on their heterotypic biofilms indicated a time-dependent shift in the interactions. Initially, the differing oxygen preferences of the two organisms allow *C. albicans* to dominate the biofilm. Yeast cells, particularly in hyphal form, colonize the surface aggressively during the early phase. After 24-48 hours, bacterial growth accelerates as *C. albicans* consumes oxygen, creating favorable anaerobic conditions for *P. gingivalis*. At 48 hours, *P. gingivalis* proliferation becomes prominent, accompanied by the production of virulence factors such as gingipains. These factors reshape the biofilm environment, inhibiting fungal growth and promoting detachment of fungal cells. These dynamic benefits both organisms: *P. gingivalis* thrives in optimal conditions, while *C. albicans* can more easily colonize new sites (Karkowska-Kuleta et al., 2018; Bartnicka et al., 2019).

Studies investigating pathogen-host interactions are conducted at various levels of organizational complexity, mostly with 2D models simulating host surfaces that engage with these pathogens. Although 2D models are cost-effective and simpler to use, their lack of structural and functional complexity limits their utility in studies requiring physiological relevance. By contrast, 3D epithelial models offer a closer approximation of *in vivo* conditions, providing detailed insights into pathogen invasion, biofilm formation, barrier integrity, immune responses, cytokine signaling, and inflammation.

Our study included two colonization models: with direct contact, where pathogens were placed in direct interaction with epithelial cells for 24 h, and model of epithelial cells exposed to the biofilm supernatant collected after 48 hours of biofilm development. The 3D structure and barrier function of epithelial cell lines such as BEAS-2B, HAE and Calu-3 undergo significant changes upon microbial infection. These alterations are influenced by each cell line’s unique characteristics, including their barrier properties and the molecules they produce.

Our research demonstrated distinct effects of *C. albicans* and *P. gingivalis* biofilms on the metabolic activity of epithelial cells. The homotypic biofilm formed by *C. albicans* caused a significant reduction in mitochondrial activity, consistent with its strong metabolic demands and ability to exploit the epithelial environment. This time-dependent reduction highlights the aggressive nature of *C. albicans* in disrupting host metabolism, likely through the secretion of extracellular enzymes and metabolic byproducts. For example, *C. albicans* biofilms are known to secrete hydrolytic enzymes — proteinases, phospholipases, and lipases — that facilitate epithelial penetration and nutrient acquisition. Among these, secreted aspartic proteinases (Saps) contribute to epithelial damage, fungal virulence, neutrophil recruitment, and pro-inflammatory cytokine production (Naglik et al., 2003; Bras et al., 2024). Phospholipases disrupt host cell membranes, while lipases enhance fungal virulence in systemic candidiasis models (Leidich et al., 1998; Hube et al., 2000; Lopes and Lionakis, 2022). Moreover, *C. albicans* in homotypic biofilm can produce metabolic byproducts such as ethanol and acetaldehyde, which disrupt mitochondrial function and induce oxidative stress (Alnuaimi et al., 2016). These findings are consistent with the time-dependent metabolic decline observed in our study, highlighting the aggressive nature of *C. albicans* biofilms under aerobic conditions.

Additionally, candidalysin, a peptide toxin secreted by *C. albicans*, likely contributes to the observed reduction in mitochondrial activity. Candidalysin is known to form pores in epithelial membranes, leading to ion dysregulation and mitochondrial dysfunction (Moyes et al., 2016; Naglik et al., 2019). This toxin has been demonstrated to trigger mitochondrial depolarization, which disrupts metabolic activity and exacerbates cellular stress in epithelial cells (Ho et al., 2020). Its involvement in our study may partially explain the pronounced effects of *C. albicans* biofilms on epithelial metabolism, especially in the context of mixed biofilms.

In contrast, the homotypic biofilm formed by *P. gingivalis*, an obligate anaerobe, resulted in only a moderate decrease in mitochondrial activity. This observation aligns with its preference for anaerobic conditions, as the oxygenated environment of lung epithelial cells may limit its metabolic impact. Other studies demonstrated that *P. gingivalis* biofilms in oxygenated environments are less metabolically active and rely on synergistic interactions with other microbes for survival (Hajishengallis and Lamont, 2014). This is consistent with our observation that mixed-species biofilms (*C. albicans* + *P. gingivalis*) exhibit similar effects on mitochondrial activity as *C. albicans* alone, suggesting that *C. albicans* creates microaerophilic conditions that support *P. gingivalis* growth.

Interestingly, in mixed bacterial biofilms involving *P. gingivalis* and other oral pathogens (e.g., *Fusobacterium nucleatum* or *Treponema denticola*), other virulence factors besides gingipains have been shown to play a role. For instance, *P. gingivalis* secretes extracellular vesicles (EVs) containing outer membrane proteins such as fimbriae, LPS, and proteases. These EVs can disrupt mitochondrial activity by inducing host inflammatory responses and mitochondrial dysfunction (Zhao et al., 2024). Such factors highlight the complex interplay within mixed-species biofilms, where *P. gingivalis* amplifies host cell stress more effectively in combination with other pathogens.

Although the exposure of epithelial cells to supernatants derived from heterotypic cultures during the period of intense gingipain production clearly enhances the effects of co-infection on host cells, this observation underlines the synergistic impact of such interactions. On the other hand, the gingipain-null mutant (ΔKΔRAB) showed reduced virulence compared to the wild-type W83 strain, as evidenced by its minimal effect on mitochondrial activity. This observation agrees with studies demonstrated that gingipains are crucial for *P. gingivalis* pathogenicity, playing roles in nutrient acquisition and immune modulation (Grenier et al., 2003; Chen et al., 2023). Our findings emphasize the synergistic relationship between these pathogens, wherein *C. albicans* may facilitate *P. gingivalis* colonization and survival under aerobic conditions. These results also reinforce the critical role of gingipains in disrupting host cell metabolism, particularly when interacting with *C. albicans*.

The enhanced penetration of *C. albicans* into epithelial barriers in the presence of *P. gingivalis* reflects the cooperative interactions between these pathogens. Our results demonstrated that co-culture with *P. gingivalis* or its supernatants significantly reduces the distance between *C. albicans* cells and the basolateral side of the epithelium, although the increase in permeability is most pronounced in BEAS-2B cells and slightly less prominent in HAE and Calu-3 cells. Nonetheless, this change is statistically significant for all tested cell types. This variation in permeability may result from the distinct properties of the epithelial models used. Primary HAE cells cultured at ALI develop a pseudostratified epithelium with ciliated and goblet cells and possess a robust barrier function due to well-formed tight junctions. In contrast, Calu-3 cells form polarized monolayers with distinct apical and basolateral surfaces, presenting a tight junction architecture that provides an intermediate barrier function (Stewart et al., 2012; He et al., 2021). BEAS-2B cells, while suitable for air-liquid interface models, typically exhibit a less robust barrier compared to HAE and Calu-3 cells. Interestingly, supernatants from the ΔKΔRAB strain of *P. gingivalis*, which lacks gingipain activity, caused minimal disruption to epithelial cells. However, when combined with *C. albicans*, even the ΔKΔRAB biofilm supernatants contributed to increased epithelial permeability, albeit to a lesser extent than those containing the virulent W83 strain. This highlights the importance of gingipains as critical virulence factors in disrupting epithelial barriers and emphasizes the synergistic role of *C. albicans* in enhancing *P. gingivalis* pathogenicity. Additionally, *P. gingivalis* fimbriae (FimA) have been shown to enhance the adhesion and invasion by disrupting epithelial adhesion complexes. Moreover, fimbriae increase epithelial permeability, promoting the penetration of infecting organisms (Yilmaz, 2008; Enersen et al., 2013).

Candidalysin also likely plays a critical role in facilitating this process by destabilizing epithelial membranes, triggering cellular stress and inducing necrotic cell death (Moyes et al., 2016; Blagojevic et al., 2021). Furthermore, candidalysin facilitates fungal translocation across epithelial barriers, as demonstrated by Allert et al., 2018. This cytotoxic activity, combined with gingipain-mediated disruption of tight junction (TJ) proteins, creates an environment highly conducive to fungal invasion. The degradation of tight junction (TJ) proteins by biofilm supernatants is a hallmark of epithelial barrier disruption (Furuse and Takai, 2021). In our study, the most pronounced reduction in TJ protein levels was observed with heterotypic biofilm supernatants, which aligns with findings showing that bacterial proteases degrade TJ proteins (Amano, 2007; Andrian et al., 2004; Tribble and Lamont, 2010; Guo et al., 2018). Interestingly, supernatants from the ΔKΔRAB mutant had minimal impact on TJ proteins, confirming that gingipains are critical mediators of TJ disruption. However, the addition of *C. albicans* to ΔKΔRAB biofilms enhanced TJ degradation, suggesting that fungal enzymes partially compensate for the absence of gingipains. This finding mirrors the work demonstrating that *C. albicans* secreted aspartyl proteases (SAPs) can degrade TJ proteins and increase epithelial permeability (Dalle et al., 2010).

The increased epithelial permeability observed following treatment with biofilm supernatants can be attributed to the degradation of key tight junction (TJ) proteins, including ZO-1 and E-cadherin, as adherens junctions protein (Nonaka et al., 2022; Carroll and Maharshak, 2013). Western blot and immunofluorescence analyses revealed a significant reduction in ZO-1 levels following exposure to supernatants from mixed-species biofilms, with nearly complete loss observed in CA+W83 treatments. E-cadherin levels were also diminished, further compromising the structural integrity of the epithelial barrier. These findings underscore the critical role of biofilm-derived secreted factors in weakening epithelial defense mechanisms.

Moreover, the mixed supernatants appeared to affect additional components of the epithelial barrier, such as β-tubulin IV, which is critical for cilia formation. The observed reduction in β-tubulin IV suggests that the heterotypic biofilm supernatants may disrupt mucociliary clearance mechanisms, further impairing epithelial defenses against inhaled pathogens. Similar results reported that bacterial biofilms impair mucociliary clearance, leading to reduced epithelial defense mechanisms (Kinane et al. 2012; Chegini et al., 2023). This effect, combined with the degradation of TJ proteins, demonstrates the multifaceted disruption of epithelial barrier integrity by *C. albicans* and *P. gingivalis* biofilms and their secreted factors. The findings have significant implications for understanding the mechanisms underlying polymicrobial infections and their role in chronic inflammatory diseases.

Of particular interest was the response of Calu-3 cells, which are widely used to model the epithelial behavior, particularly in the context of mucin production. Mucins, a family of high molecular weight O-glycosylated proteins, play a crucial role in protecting the respiratory tract from viral and bacterial infections (Chatterjee et al., 2023). Among these, the gel-forming mucin MUC5AC is critical for eliminating pathogens through mucociliary clearance. In our model, a significant increase in mucin production was observed following exposure to the supernatant of the heterotypic biofilm. This suggests heightened epithelial cell preparedness for microbial invasion, likely stimulated by factors released into the environment by the interacting pathogens. These findings contrast with the known effects of *P. gingivalis*, whose gingipains have been shown to degrade the inner protective mucus layer through specific cleavage of mucins. This process is modulated by site-specific O-glycosylation, which influences gingipain activity and its impact on mucin integrity (Van Der Post et al., 2013). The interplay between enhanced mucin production and potential degradation by gingipains warrants further investigation to understand the dual effects of these polymicrobial interactions.

From the perspective of *Candida*, varying outcomes can be anticipated depending on the progression of the infection. On one side, mucins influence the morphology and function of *C. albicans* by downregulating the expression of genes associated with adherence, filamentation, and biofilm formation (Kavanaugh et al., 2014) Conversely, *C. albicans* has evolved strategies to overcome the protective mucus barrier. It can attach to mucins through a pH-independent mechanism involving hydrophobic interactions (de Repentigny et al., 2000). Following adhesion, *C. albicans* disrupts the mucus layer—particularly in the gastrointestinal tract—by secreting enzymes that degrade mucus components. Among these, secreted aspartyl protease 2 plays a crucial role in mucus penetration by forming pores in the mucus layer (Colina et al., 1996; Basmaciyan et al., 2019).

Our results also shed light on the inflammatory and oxidative stress responses elicited by these pathogens. The dual-species biofilm of *C. albicans* and *P. gingivalis* was associated with reduced IL-8 secretion compared to *C. albicans* alone. This attenuation likely reflects immune evasion strategies employed by the pathogens, particularly *P. gingivalis*, which is known to modulate host immune responses through gingipains. However, *C. albicans* remained the dominant driver of pro-inflammatory responses, as evidenced by its strong induction of IL-8 secretion, a finding consistent with previous studies which investigated homotypic fungal infection of epithelial cells (Dongari-Bagtzoglou and Kashleva, 2003). Moreover, candidalysin is a key contributor to the inflammatory response observed in fungal biofilms (Richardson et al., 2018; Naglik et al., 2019), and triggers the activation of MAPK and NF-κB pathways, leading to the production of pro-inflammatory cytokines such as IL-8 and IL-1β.

In contrast, *P. gingivalis* biofilms suppressed IL-8 secretion by degrading cytokines and their receptors reflecting its immune-modulatory strategies (Mikolajczyk-Pawlinska et al., 1998; Palm et al., 2013; Hajishengallis et al., 2012). These findings highlight the complex interplay between fungal and bacterial biofilms in modulating inflammation, which could have implications for chronic infections.

Oxidative stress, a critical factor in epithelial damage, was assessed through the expression of HO-1 and apoptotic markers. In our model, HO-1 levels were significantly reduced in cells exposed to *C. albicans* alone or in combination with *P. gingivalis*. This suppression aligns with findings presenting that *C. albicans* downregulates the p38-Nrf2-HO-1 pathway, leading to diminished antioxidant defenses (Ren et al., 2021). Such suppression likely exacerbates oxidative stress, contributing to reduced mitochondrial function and cell viability, as observed in our study.

The synergistic effect of the mixed-species biofilm is further supported by increased pro-apoptotic Bax/Bcl-xL ratios, indicating heightened mitochondrial membrane permeabilization and apoptosis. The role of Bax in mitochondrial dysfunction and the execution of apoptosis, coupled with reduced Bcl-xL levels, aligns with established mechanisms of cell death under oxidative stress (Renault et al., 2017). Bax α exists in a latent form and is activated through conformational changes following the induction of apoptosis, while the β isoform is tightly regulated by proteasomal degradation (Fu et al., 2009). The disappearance of the β isoform, along with enhanced Bax α expression, may result from the increased degradation of the former and the activation of the latter. Enhanced Bax activation has been demonstrated under hypoxic conditions, which are known to promote apoptosis (Mikhailov et al., 2003). These findings underscore the capacity of polymicrobial biofilms to intensify oxidative damage and apoptosis, further compromising epithelial integrity. Similar findings were reported for a mixed species infection involving *P. gingivalis* and the influenza A virus H1N1, which exacerbated epithelial cell damage (Chen et al., 2018).

The survival experiments using *Galleria mellonella* larvae provided a translational perspective on these findings. Larvae injected with supernatants from mixed-species biofilms exhibited reduced survival, particularly when treated with low doses of levofloxacin. However, high-dose levofloxacin significantly improved larval survival, indicating that targeting *P. gingivalis* virulence factors, such as gingipains, can mitigate its pathogenicity (Bras et al., 2025). This phenomenon is likely driven by the increased production of bacterial outer membrane vesicles (OMVs), which serve as the primary source of gingipains (Okamura et al., 2021) and can also neutralize the antibiotic’s effect by acting as “decoys,” either by binding to or encapsulating the antibiotic (Jiang et al., 2024). These results emphasize the importance of selecting optimal drug concentrations to avoid unintended enhancement of microbial virulence. The antibiotic-induced increase in gingipain activity at suboptimal concentrations serves as a cautionary example of how partial suppression of bacterial populations can lead to compensatory pathogenic mechanisms, potentially undermining treatment success. Understanding such interactions is crucial for designing combination therapy regimens that not only target pathogen viability but also mitigate microbial adaptations that could compromise therapeutic efficacy. The clinical relevance of these findings is particularly evident in the context of polymicrobial infections, where interactions between co-infecting bacterial and fungal species can significantly alter disease progression and treatment outcomes.

## 5 Conclusions

**Figure 8:**
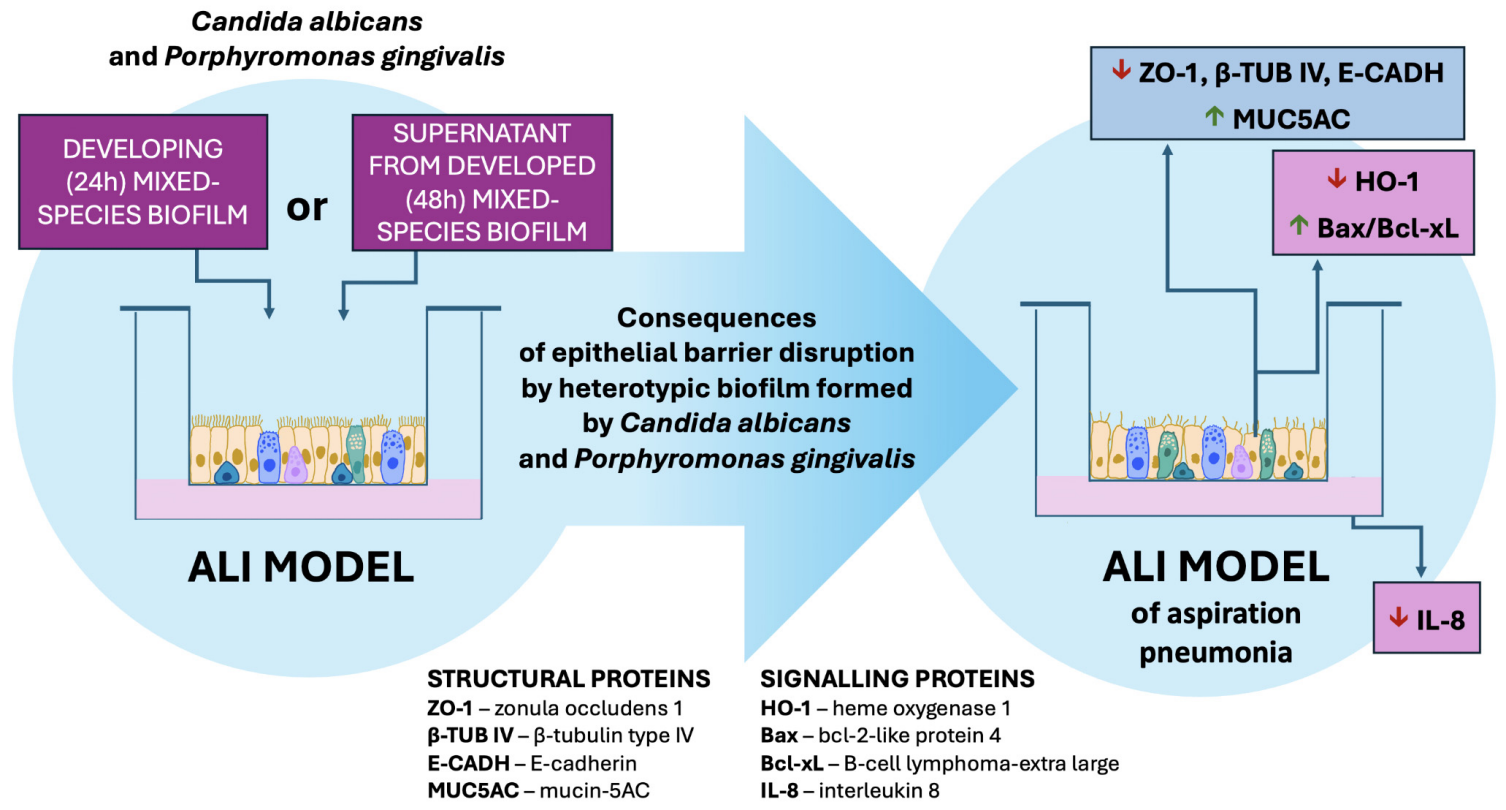
The influence of a mixed biofilm of *Candida albicans* and *Porphyromonas gingivalis* on the integrity of epithelial cells in the ALI model of aspiration pneumonia. The heterotypic biofilm formed by *C. albicans* and *P. gingivalis*, along with their secreted factors, has the potential to disrupt epithelial structural integrity and signaling proteins. Additionally, this biofilm may attenuate the pro-inflammatory response of host cells while promoting apoptosis in epithelial cells.

Our study provides compelling evidence of the complex and synergistic interactions between *C. albicans* and *P. gingivalis* that compromise epithelial barrier function (**Figure 8**). The findings underscore the significant impact of mixed-species biofilms, where the cooperative mechanisms between these pathogens amplify epithelial damage. Key achievements of this study include:

- Demonstrating the enhanced virulence of *C. albicans* and *P. gingivalis* in mixed biofilms compared to single-species biofilms.
- Highlighting the role of secreted factors, such as gingipains and candidalysin, in disrupting epithelial integrity and promoting inflammatory and oxidative stress responses.
- Revealing the ability of *C. albicans* to create a supportive niche that facilitates the survival and pathogenicity of *P. gingivalis* in aerobic environments.

These results highlight the need to consider polymicrobial interactions in both research and clinical settings, as they represent a critical factor in the pathogenesis of chronic infections. Targeting the cooperative mechanisms of these pathogens may provide new therapeutic avenues to mitigate epithelial damage and improve outcomes in polymicrobial infections.

## Data Availability

The datasets presented in this study can be found in online repositories. The names of the repository/repositories and accession number(s) can be found in the article/**Supplementary Material**.

## References

[B1] Allert S. Förster T. M. Svensson C. M. Richardson J. P. Pawlik T. Hebecker B.. (2018). *Candida albicans*-induced epithelial damage mediates translocation through intestinal barriers. mBio 9, e00915-18. doi: 10.1128/mBio.00915-18 29871918 PMC5989070

[B2] Almirall J. Boixeda R. de la Torre M. C. Torres A. (2021). Aspiration pneumonia: A renewed perspective and practical approach. Respir. Med. 185, 106485. doi: 10.1016/j.rmed.2021.106485 34087609

[B3] Alnuaimi A. D. Ramdzan A. N. Wiesenfeld D. O’Brien-Simpson N. M. Kolev S. D. Reynolds E. C.. (2016). *Candida* virulence and ethanol-derived acetaldehyde production in oral cancer and non-cancer subjects. Oral. Dis. 22, 805–814. doi: 10.1111/odi.12565 27495361

[B4] Alonso-Monge R. Cortés-Prieto I. Román E. Pla J. (2024). Morphogenetic transitions in the adaptation of *Candida albican*s to the mammalian gut. Microbes Infection 26, 105253. doi: 10.1016/j.micinf.2023.105253 37977323

[B5] Amano A. (2007). Disruption of epithelial barrier and impairment of cellular function by *Porphyromonas gingivalis* . Front. Bioscience. 12, 3965–3974. doi: 10.2741/2363 17485350

[B6] Amano A. Takeuchi H. Furuta N. (2010). Outer membrane vesicles function as offensive weapons in host-parasite interactions. Microbes Infection 12, 791–798. doi: 10.1016/j.micinf.2010.05.008 20685339

[B7] Andrian E. Grenier D. Rouabhia M. (2004). *In vitro* models of tissue penetration and destruction by *Porphyromonas gingivalis* . Infection Immun. 72, 4689–4698. doi: 10.1128/IAI.72.8.4689-4698.2004 PMC470627 15271930

[B8] Bartnicka D. Karkowska-Kuleta J. Zawrotniak M. Satała D. Michalik K. Zielinska G.. (2019). Adhesive protein-mediated cross-talk between *Candida albicans* and *Porphyromonas gingivalis* in dual species biofilm protects the anaerobic bacterium in unfavorable oxic environment. Sci. Rep. 9, 4376. doi: 10.1038/s41598-019-40771-8 30867500 PMC6416349

[B9] Basmaciyan L. Bon F. Paradis T. Lapaquette P. Dalle F. (2019). *Candida albicans* interactions with the host: crossing the intestinal epithelial barrier. Tissue barriers 7, 1612661. doi: 10.1080/21688370.2019.1612661 PMC6619947 31189436

[B10] Benedyk M. Mydel P. M. Delaleu N. Płaza K. Gawron K. Milewska A.. (2016). Gingipains: critical factors in the development of aspiration pneumonia caused by *porphyromonas gingivalis* . J. Innate Immun. 8, 185–198. doi: 10.1159/000441724 26613585 PMC4801669

[B11] Blagojevic M. Camilli G. Maxson M. Hube B. Moyes D. L. Richardson J. P.. (2021). Candidalysin triggers epithelial cellular stresses that induce necrotic death. Cell. Microbiol. 23, e13371. doi: 10.1111/cmi.13371 34085369 PMC8460601

[B12] Bostanci N. Belibasakis G. N. (2012). *Porphyromonas gingivalis:* An invasive and evasive opportunistic oral pathogen. FEMS Microbiol. Lett. 333, 1–9. doi: 10.1111/j.1574-6968.2012.02579.x 22530835

[B13] Bras G. Satala D. Juszczak M. Kulig K. Wronowska E. Bednarek A.. (2024). Secreted aspartic proteinases: key factors in *candida* infections and host-pathogen interactions. Int. J. Mol. Sci. 25, 4775. doi: 10.3390/ijms25094775 38731993 PMC11084781

[B14] Bras G. Wronowska E. Gonzalez-Gonzalez M. Juszczak M. Surowiec M. Sidlo W.. (2025). The efficacy of antimicrobial therapies in the treatment of mixed biofilms formed between *Candida albicans* and *Porphyromonas gingivalis* during epithelial cell infection in the aspiration pneumonia model. Med. Microbiol. Immunol. 214, 8. doi: 10.1007/s00430-025-00818-2 39903321 PMC11794384

[B15] Carlisle M. D. Srikantha R. N. Brogden K. A. (2009). Degradation of human α- And β-defensins by culture supernatants of *Porphyromonas gingivalis* strain 381. J. Innate Immun. 1, 118–122. doi: 10.1159/000181015 20375570 PMC7312840

[B16] Carroll I. M. Maharshak N. (2013). Enteric bacterial proteases in inflammatory bowel diseasepathophysiology and clinical implications. World J. Gastroenterol. 19, 7531–7543. doi: 10.3748/wjg.v19.i43.7531 24431894 PMC3837251

[B17] Chatterjee M. Huang L. Z. X. Mykytyn A. Z. Wang C. Lamers M. M. Westendorp B.. (2023). Glycosylated extracellular mucin domains protect against SARS-CoV-2 infection at the respiratory surface’. PloS Pathog. 19, e1011571. doi: 10.1371/journal.ppat.1011571 37561789 PMC10464970

[B18] Chegini Z. Noei M. Hemmati J. Arabestani M. R. Shariati A. (2023). The destruction of mucosal barriers, epithelial remodeling, and impaired mucociliary clearance: possible pathogenic mechanisms of *Pseudomonas aeruginosa* and *Staphylococcus aureus* in chronic rhinosinusitis. Cell Communication Signaling. 21, 306. doi: 10.1186/s12964-023-01347-2 37904180 PMC10614382

[B19] Chen W. A. Dou Y. Fletcher H. M. Boskovic D. S. (2023). Local and systemic effects of *porphyromonas gingivalis* infection. Microorganisms 11, 470. doi: 10.3390/microorganisms11020470 36838435 PMC9963840

[B20] Chen Y. Zhou R. Yi Z. Li Y. Fu Y. Zhang Y.. (2018). *Porphyromonas gingivalis* induced inflammatory responses and promoted apoptosis in lung epithelial cells infected with H1N1 via the Bcl-2/Bax/Caspase-3 signaling pathway. Mol. Med. Rep. 18, 97–104. doi: 10.3892/mmr.2018.8983 29750299 PMC6059728

[B21] Chow E. W. L. Pang L. M. Wang Y. (2021). From Jekyll to Hyde: The yeast–hyphal transition of *Candida albicans* . Pathogens. 10, 859. doi: 10.3390/pathogens10070859 34358008 PMC8308684

[B22] Colina A. R. Aumont F. Deslauriers N. Belhumeur P. De Repentigny L. (1996). Evidence for degradation of gastrointestinal mucin by *Candida albicans* secretory aspartyl proteinase’. Infection Immun. 64, 4514–4519. doi: 10.1128/iai.64.11.4514-4519.1996 PMC174406 8890200

[B23] Cutler C. W. Kalmar J. R. Genco C. A. (1995). Pathogenic strategies of the oral anaerobe, *Porphyromonas gingivalis* . Trends Microbiol. 3, 45–51. doi: 10.1016/S0966-842X(00)88874-5 7728384

[B24] Dalle F. Wächtler B. L’Ollivier C. Holland G. Bannert N. Wilson D.. (2010). Cellular interactions of *Candida albicans* with human oral epithelial cells and enterocytes. Cell. Microbiol. 12, 248–271. doi: 10.1111/j.1462-5822.2009.01394.x 19863559

[B25] De Diego I. Veillard F. Sztukowska M. N. Guevara T. Potempa B. Pomowski A.. (2014). Structure and mechanism of cysteine peptidase gingipain K (Kgp), a major virulence factor of *Porphyromonas gingivalis* . J. Biol. Chem. 289, 32291–32302. doi: 10.1074/jbc.M114.602052 25266723 PMC4231702

[B26] de Jongh C. A. Bikker F. J. de Vries T. J. Werner A. Gibbs S. Krom B. P. (2024). *Porphyromonas gingivalis* interaction with *Candida albicans* allows for aerobic escape, virulence and adherence. Biofilm 7, 100172. doi: 10.1016/j.bioflm.2023.100172 38226024 PMC10788424

[B27] de Repentigny L. Aumont F. Bernard K. Belhumeur P. (2000). Characterization of binding of *Candida albicans* to small intestinal mucin and its role in adherence to mucosal epithelial cells. Infection Immun. 68, 3172–3179. doi: 10.1128/IAI.68.6.3172-3179.2000 PMC97555 10816460

[B28] Dhanisha S. S. Guruvayoorappan C. Drishya S. Abeesh P. (2018). Mucins: Structural diversity, biosynthesis, its role in pathogenesis and as possible therapeutic targets. Crit. Rev. Oncology/Hematology. 122, 98–122. doi: 10.1016/j.critrevonc.2017.12.006 29458795

[B29] Dominy S. S. Lynch C. Ermini F. Benedyk M. Marczyk A. Konradi A.. (2019). *Porphyromonas gingivalis* in Alzheimer’s disease brains: Evidence for disease causation and treatment with small-molecule inhibitors’. Sci. Adv. 5, eaau3333. doi: 10.1126/sciadv.aau3333 30746447 PMC6357742

[B30] Dongari-Bagtzoglou A. Kashleva H. (2003). *Candida albican*s triggers interleukin-8 secretion by oral epithelial cells. Microbial Pathogenesis 34, 169–177. doi: 10.1016/S0882-4010(03)00004-4 12668140

[B31] Enersen M. Nakano K. Amano A. (2013). *Porphyromonas gingivalis* fimbriae. J. Oral. Microbiol. 5. doi: 10.3402/jom.v5i0.20265 PMC3647041 23667717

[B32] Fandiño-Devia E. Brankiewicz A. Santa-González G. A. Guevara-Lora I. Manrique-Moreno M. (2024). Comparative study of the potential cell-penetrating peptide ΔM4 on apoptosis cell signaling in A375 and A431 cancer cell lines. Pharmaceutics 16, 775. doi: 10.3390/pharmaceutics16060775 38931896 PMC11207241

[B33] Feller L. Khammissa R. A. G. Chandran R. Altini M. Lemmer J. (2014). Oral candidosis in relation to oral immunity. J. Oral. Pathol. Med. 43, 563–569. doi: 10.1111/jop.12120 24118267

[B34] Fox E. P. Cowley E. S. Nobile C. J. Hartooni N. Newman D. K. Johnson A. D. (2014). Anaerobic bacteria grow within candida albicans biofilms and induce biofilm formation in suspension cultures. Curr. Biol. 24, 2411–2416. doi: 10.1016/j.cub.2014.08.057 25308076 PMC4252622

[B35] Fu N. Y. Sukumaran S. K. Kerk S. Y. Yu V. C. (2009). Baxβ: A constitutively active human Bax isoform that is under tight regulatory control by the proteasomal degradation mechanism. Mol. Cell 33, 15–29. doi: 10.1016/j.molcel.2008.11.025 19150424

[B36] Furuse M. Takai Y. (2021). Recent advances in understanding tight junctions. Faculty Rev. 10. doi: 10.12703/r/10-18 PMC7946388 33718935

[B37] Gomes C. C. Guimarães L. S. Pinto L. C. C. Camargo G.A.D.C.G. Valente M. I. B. Sarquis M. I. D. M. (2017). Investigations of the prevalence and virulence of *Candida albicans* in periodontal and endodontic lesions in diabetic and normoglycemic patients. J. Appl. Oral. Sci. 25, 274–281. doi: 10.1590/1678-7757-2016-0432 28678946 PMC5482250

[B38] Gow N. A. R. Yadav B. (2017). Microbe profile: *Candida albicans*: A shape-changing, opportunistic pathogenic fungus of humans. Microbiol. (United Kingdom) 163, 1145–1147. doi: 10.1099/mic.0.000499 28809155

[B39] Grenier D. Roy S. Chandad F. Plamondon P. Yoshioka M. Nakayama K.. (2003). Effect of inactivation of the Arg- and/or Lys-gingipain gene on selected virulence and physiological properties of *Porphyromonas gingivalis* . Infection Immun. 71, 4742–4748. doi: 10.1128/IAI.71.8.4742-4748.2003 PMC166032 12874356

[B40] Guo W. Wang P. Liu Z. H. Ye P. (2018). Analysis of differential expression of tight junction proteins in cultured oral epithelial cells altered by *Porphyromonas gingivalis*, *Porphyromonas gingivalis* lipopolysaccharide, and extracellular adenosine triphosphate. Int. J. Oral. Sci. 10, e8. doi: 10.1038/ijos.2017.51 29319048 PMC5795020

[B41] Hajishengallis G. Darveau R. P. Curtis M. A. (2012). The keystone-pathogen hypothesis. Nat. Rev. Microbiol. 10, 717–725. doi: 10.1038/nrmicro2873 22941505 PMC3498498

[B42] Hajishengallis G. Lamont R. J. (2014). Breaking bad: Manipulation of the host response by *Porphyromonas gingivalis* . Eur. J. Immunol. 44, 328–338. doi: 10.1002/eji.201344202 24338806 PMC3925422

[B43] Hamada S. Amano A. Kimura S. Nakagawa I. Kawabata S. Morisaki I. (1998). The importance of fimbriae in the virulence and ecology of some oral bacteria. Oral. Microbiol. Immunol. 13, 129–138. doi: 10.1111/j.1399-302X.1998.tb00724.x 10093527

[B44] He R. W. Braakhuis H. M. Vandebriel R. J. Staal Y. C. M. Gremmer E. R. Fokkens P. H. B.. (2021). Optimization of an air-liquid interface *in vitro* cell co-culture model to estimate the hazard of aerosol exposures. J. Aerosol Sci. 153, 105703. doi: 10.1016/j.jaerosci.2020.105703 33658726 PMC7874005

[B45] Ho J. Wickramasinghe D. N. Nikou S. A. Hube B. Richardson J. P. Naglik J. R. (2020). Candidalysin is a potent trigger of alarmin and antimicrobial peptide release in epithelial cells. Cells 9, 699. doi: 10.3390/cells9030699 32178483 PMC7140650

[B46] Hube B. Stehr F. Bossenz M. Mazur A. Kretschmar M. Schäfer W. (2000). Secreted lipases of *Candida albicans*: Cloning, characterisation and expression analysis of a new gene family with at least ten members. Arch. Microbiol. 174, 362–374. doi: 10.1007/s002030000218 11131027

[B47] Huffnagle G. B. Noverr M. C. (2013). The emerging world of the fungal microbiome. Trends Microbiol. 21, 334–341. doi: 10.1016/j.tim.2013.04.002 23685069 PMC3708484

[B48] Jakiela B. Brockman-Schneider R. Amineva S. Lee W. M. Gern J. E. (2008). Basal cells of differentiated bronchial epithelium are more susceptible to rhinovirus infection. Am. J. Respir. Cell Mol. Biol. 38, 517–523. doi: 10.1165/rcmb.2007-0050OC 18063839 PMC2358970

[B49] Jiang B. Lai Y. Xiao W. Zhong T. Liu F. Gong J.. (2024). Microbial extracellular vesicles contribute to antimicrobial resistance. PloS Pathog. 20, e1012143. doi: 10.1371/journal.ppat.1012143 38696356 PMC11065233

[B50] Kaminishi H. Miyaguchi H. Tamaki T. Suenaga N. Hisamatsu M. Mihashi I.. (1995). Degradation of humoral host defense by *Candida albicans* proteinase. Infection Immun. 63, 984–988. doi: 10.1128/iai.63.3.984-988.1995 PMC173099 7868271

[B51] Kamio N. Hayata M. Tamura M. Tanaka H. Imai K. (2021). *Porphyromonas gingivalis* enhances pneumococcal adhesion to human alveolar epithelial cells by increasing expression of host platelet-activating factor receptor. FEBS Lett. 595, 1604–1612. doi: 10.1002/1873-3468.14084 33792027

[B52] Karkowska-Kuleta J. Bartnicka D. Zawrotniak M. Zielinska G. Kieronska A. Bochenska O.. (2018). The activity of bacterial peptidylarginine deiminase is important during formation of dual-species biofilm by periodontal pathogen *Porphyromonas gingivalis* and opportunistic fungus *Candida albicans* . Pathog. Dis. 76, fty033. doi: 10.1093/femspd/fty033 29668945 PMC6251568

[B53] Kavanaugh N. L. Zhang A. Q. Nobile C. J. Johnson A. D. Ribbeck K. (2014). Mucins suppress virulence traits of *Candida albicans* . mBio 5, e01911. doi: 10.1128/mBio.01911-14 25389175 PMC4235211

[B54] Kikuchi T. Shively J. D. Foley J. S. Drazen J. M. Tschumperlin D. J. (2004). Differentiation-dependent responsiveness of bronchial epithelial cells to IL-4/13 stimulation. Am. J. Physiol. - Lung Cell. Mol. Physiol. 287, L119–26. doi: 10.1152/ajplung.00365.2003 15020299

[B55] Kinane J. A. Benakanakere M. R. Zhao J. Hosur K. B. Kinane D. F. (2012). *Porphyromonas gingivalis* influences actin degradation within epithelial cells during invasion and apoptosis. Cell. Microbiol. 14, 1085–1096. doi: 10.1111/j.1462-5822.2012.01780.x 22381126

[B56] Kuboniwa M. Amano A. Hashino E. Yamamoto Y. Inaba H. Hamada N.. (2009). Distinct roles of long/short fimbriae and gingipains in homotypic biofilm development by *Porphyromonas gingivalis* . BMC Microbiol. 9, 105. doi: 10.1186/1471-2180-9-105 19470157 PMC2697998

[B57] Kulig K. Kowalik K. Surowiec M. Karnas E. Barczyk-Woznicka O. Zuba-Surma E.. (2024). Isolation and characteristics of extracellular vesicles produced by probiotics: Yeast *Saccharomyces boulardii* CNCM I-745 and Bacterium *Streptococcus salivarius* K12. Probiotics Antimicrobial Proteins 16, 936–948. doi: 10.1007/s12602-023-10085-3 37209320 PMC11126510

[B58] Lacroix G. Koch W. Ritter D. Gutleb A. C. Larsen S. T. Loret T.. (2018). Air-Liquid Interface *in vitro* models for respiratory toxicology research: Consensus workshop and recommendations. Appl. In Vitro Toxicol. 4, 91–106. doi: 10.1089/aivt.2017.0034 PMC7500038 32953944

[B59] Lamont R. J. Jenkinson H. F. (1998). Life below the gum line: Pathogenic mechanisms of *Porphyromonas gingivalis* . Microbiol. Mol. Biol. Rev. 62, 1244–1263. doi: 10.1128/mmbr.62.4.1244-1263.1998 9841671 PMC98945

[B60] Leidich S. D. Ibrahim A. S. Fu Y. Koul A. Jessup C. Vitullo J.. (1998). Cloning and disruption of caPLB1, a phospholipase B gene involved in the pathogenicity of *Candida albicans* . J. Biol. Chem. 273, 26078–26086. doi: 10.1074/jbc.273.40.26078 9748287

[B61] Lenart M. Górecka M. Bochenek M. Barreto-Duran E. Szczepański A. Gałuszka-Bulaga A.. (2023). SARS-CoV-2 infection impairs NK cell functions via activation of the LLT1-CD161 axis. Front. Immunol. 14. doi: 10.3389/fimmu.2023.1123155 PMC10242091 37287972

[B62] Loboda A. Damulewicz M. Pyza E. Jozkowicz A. Dulak J. (2016). Role of Nrf2/HO-1 system in development, oxidative stress response and diseases: an evolutionarily conserved mechanism. Cell. Mol. Life Sci. 73, 3221–3247. doi: 10.1007/s00018-016-2223-0 27100828 PMC4967105

[B63] Lopes J. P. Lionakis M. S. (2022). Pathogenesis and virulence of *Candida albicans* . Virulence 13, 89–121. doi: 10.1080/21505594.2021.2019950 34964702 PMC9728475

[B64] Matsushima K. Yang D. Oppenheim J. J. (2022). Interleukin-8: An evolving chemokine. Cytokine 153, 155828. doi: 10.1016/j.cyto.2022.155828 35247648

[B65] Mikhailov V. Mikhailova M. Degenhardt K. Venkatachalam M. A. White E. Saikumar P. (2003). Association of Bax and Bak homo-oligomers in mitochondria: Bax requirement for Bak reorganization and cytochrome c release. J. Biol. Chem. 278, 5367–5376. doi: 10.1074/jbc.M203392200 12454021

[B66] Mikolajczyk-Pawlinska J. Travis J. Potempa J. (1998). Modulation of interleukin-8 activity by gingipains from *Porphyromonas gingivalis*: Implications for pathogenicity of periodontal disease. FEBS Lett. 440, 282–286. doi: 10.1016/S0014-5793(98)01461-6 9872387

[B67] Ménard G. Rouillon A. Cattoir V. Donnio P. Y. (2021). Galleria mellonella as a Suitable Model of Bacterial Infection: Past, Present and Future. Frontiers in cellular and infection microbiology 11, 782733. doi: 10.3389/fcimb.2021.782733 35004350 PMC8727906

[B68] Moyes D. L. Wilson D. Richardson J. P. Mogavero S. Tang S. X. Wernecke J.. (2016). Candidalysin is a fungal peptide toxin critical for mucosal infection. Nature 532, 64–68. doi: 10.1038/nature17625 27027296 PMC4851236

[B69] Naglik J. R. Challacombe S. J. Hube B. (2003). *Candida albicans* secreted aspartyl proteinases in virulence and pathogenesis. Microbiol. Mol. Biol. Rev. 67, 400–428. doi: 10.1128/mmbr.67.3.400-428.2003 12966142 PMC193873

[B70] Naglik J. R. Gaffen S. L. Hube B. (2019). Candidalysin: discovery and function in *Candida albicans* infections. Curr. Opin. Microbiol. 52, 100–109. doi: 10.1016/j.mib.2019.06.002 31288097 PMC6687503

[B71] Nativel B. Couret D. Giraud P. Meilhac O. D’Hellencourt C. L. Viranaïcken W.. (2017). *Porphyromonas gingivalis* lipopolysaccharides act exclusively through TLR4 with a resilience between mouse and human. Sci. Rep. 7, 15789. doi: 10.1038/s41598-017-16190-y 29150625 PMC5693985

[B72] Neill S. Dean N. (2019). Aspiration pneumonia and pneumonitis: A spectrum of infectious/noninfectious diseases affecting the lung. Curr. Opin. Infect. Dis. 32, 152–157. doi: 10.1097/QCO.0000000000000524 30676341

[B73] Nikou S. A. Kichik N. Brown R. Ponde N. O. Ho J. Naglik J. R.. (2019). *Candida albicans* interactions with mucosal surfaces during health and disease. Pathogens 8, 53. doi: 10.3390/pathogens8020053 31013590 PMC6631630

[B74] Nonaka S. Kadowaki T. Nakanishi H. (2022). Secreted gingipains from *Porphyromonas gingivalis* increase permeability in human cerebral microvascular endothelial cells through intracellular degradation of tight junction proteins. Neurochemistry Int. 154, 105282. doi: 10.1016/j.neuint.2022.105282 35032577

[B75] O’Brien-Simpson N. M. Paolini R. A. Hoffmann B. Slakeski N. Dashper S. G. Reynolds E. C. (2001). Role of RgpA, RgpB, and Kgp proteinases in virulence of *Porphyromonas gingivalis* W50 in a murine lesion model. Infection Immun. 69, 7527–7534. doi: 10.1128/IAI.69.12.7527-7534.2001 PMC98843 11705929

[B76] O’Donnell L. E. Millhouse E. Sherry L. Kean R. Malcolm J. Nile C. J.. (2015). Polymicrobial *Candida* biofilms: friends and foe in the oral cavity. FEMS yeast Res. 15, fov077. doi: 10.1093/femsyr/fov077 26298018

[B77] Ogulur I. Pat Y. Yazici D. Ardicli S. Ardicli O. Mitamura Y.. (2024). Epithelial barrier dysfunction, type 2 immune response, and the development of chronic inflammatory diseases. Curr. Opin. Immunol. 91, 102493. doi: 10.1016/j.coi.2024.102493 39321494

[B78] Okamura H. Hirota K. Yoshida K. Weng Y. He Y. Shiotsu N.. (2021). Outer membrane vesicles of *Porphyromonas gingivalis*: Novel communication tool and strategy. Japanese Dental Sci. Rev. 57, 138–146. doi: 10.1016/j.jdsr.2021.07.003 PMC8399048 34484474

[B79] Ottaviano E. Borghi E. Giovati L. Falleni M. Tosi D. Magliani W.. (2021). Therapeutic effect of an antibody-derived peptide in a *Galleria mellonella* model of systemic candidiasis. Int. J. Mol. Sci. 22, 10904. doi: 10.3390/ijms222010904 34681564 PMC8536055

[B80] Palm E. Khalaf H. Bengtsson T. (2013). *Porphyromonas gingivalis* downregulates the immune response of fibroblasts. BMC Microbiol. 13, 155. doi: 10.1186/1471-2180-13-155 23841502 PMC3717116

[B81] Perlroth J. Choi B. Spellberg B. (2007). Nosocomial fungal infections: Epidemiology, diagnosis, and treatment. Med. Mycology 45, 321–346. doi: 10.1080/13693780701218689 17510856

[B82] Ponde N. O. Lortal L. Ramage G. Naglik J. R. Richardson J. P. (2021). *Candida albicans* biofilms and polymicrobial interactions. Crit. Rev. Microbiol. 47, 91–111. doi: 10.1080/1040841X.2020.1843400 33482069 PMC7903066

[B83] Potempa M. Potempa J. (2012). Protease-dependent mechanisms of complement evasion by bacterial pathogens. Biol. Chem. 393, 873–888. doi: 10.1515/hsz-2012-0174 22944688 PMC3488274

[B84] Potempa J. Sroka A. Imamura T. Travis J. (2005). Gingipains, the major cysteine proteinases and virulence factors of *Porphyromonas gingivalis*: Structure, function and assembly of multidomain protein complexes. Curr. Protein Pept. Sci. 4, 397–407. doi: 10.2174/1389203033487036 14683426

[B85] Rao R. (2008). Oxidative stress-induced disruption of epithelial and endothelial tight junctions. Front. Bioscience 13, 7210–7226. doi: 10.2741/3223 PMC6261932 18508729

[B86] Ren T. Zhu H. Tian L. Yu Q. Li M. (2021). *Candida albicans* infection disturbs the redox homeostasis system and induces reactive oxygen species accumulation for epithelial cell death. FEMS Yeast Res. 20, foz081. doi: 10.1093/FEMSYR/FOZ081 31769804

[B87] Renault T. T. Dejean L. M. Manon S. (2017). A brewing understanding of the regulation of Bax function by Bcl-xL and Bcl-2. Mech. Ageing Dev. 161, 201–210. doi: 10.1016/j.mad.2016.04.007 27112371

[B88] Richardson J. P. Willems H. M. E. Moyes D. L. Shoaie S. Barker K. S. Tan S. L.. (2018). Candidalysin drives epithelial signaling, neutrophil recruitment, and immunopathology at the vaginal mucosa. Infection Immun. 86, e00645–17. doi: 10.1128/IAI.00645-17 PMC5778364 29109176

[B89] Sardi J. C. O. Scorzoni L. Bernardi T. Fusco-Almeida A. M. Mendes Giannini M. J. S. (2013). *Candida* species: Current epidemiology, pathogenicity, biofilm formation, natural antifungal products and new therapeutic options. J. Med. Microbiol. 62, 10–24. doi: 10.1099/jmm.0.045054-0 23180477

[B90] Satala D. Gonzalez-Gonzalez M. Smolarz M. Surowiec M. Kulig K. Wronowska E.. (2022). The role of *Candida albicans* virulence factors in the formation of multispecies biofilms with bacterial periodontal pathogens. Front. Cell. Infection Microbiol. 11. doi: 10.3389/fcimb.2021.765942 PMC8766842 35071033

[B91] Silva S. Bicker J. Falcão A. Fortuna A. (2023). Air-liquid interface (ALI) impact on different respiratory cell cultures. Eur. J. Pharmaceutics Biopharmaceutics 184, 62–82. doi: 10.1016/j.ejpb.2023.01.013 36696943

[B92] Simpson A. Allen J. L. Chatwin M. Crawford H. Elverson J. Ewan V.. (2023). BTS clinical statement on aspiration pneumonia. Thorax 78, s3–s21. doi: 10.1136/thorax-2022-219699 36863772

[B93] Singh A. Verma R. Murari A. Agrawal A. (2014). Oral candidiasis: An overview. J. Oral. Maxillofac. Pathol. 18, 81–85. doi: 10.4103/0973-029X.141325 PMC4211245 25364186

[B94] Singh A. Wyant T. Anaya-Bergman C. Aduse-Opoku J. Brunner J. Laine M. L.. (2011). The capsule of porphyromonas gingivalis leads to a reduction in the host inflammatory response, evasion of phagocytosis, and increase in Virulence. Infection Immun. 79, 4533–4542. doi: 10.1128/IAI.05016-11 PMC3257911 21911459

[B95] Socransky S. S. Haffajee A. D. Cugini M. A. Smith C. Kent R. L. (1998). Microbial complexes in subgingival plaque. J. Clin. Periodontology 25, 134–144. doi: 10.1111/j.1600-051X.1998.tb02419.x 9495612

[B96] Stepanenko A. A. Dmitrenko V. V. (2015). Pitfalls of the MTT assay: Direct and off-target effects of inhibitors can result in over/underestimation of cell viability. Gene 574, 193–203. doi: 10.1016/j.gene.2015.08.009 26260013

[B97] Stewart C. E. Torr E. E. Mohd Jamili N. H. Bosquillon C. Sayers I. (2012). Evaluation of differentiated human bronchial epithelial cell culture systems for asthma research. J. Allergy 2012, 1–11. doi: 10.1155/2012/943982 PMC3263641 22287976

[B98] Sztukowska M. N. Dutton L. C. Delaney C. Ramsdale M. Ramage G. Jenkinson H. F.. (2018). Community development between *Porphyromonas gingivali*s and *Candida albicans* mediated by inIJ and Als3. mBio 9, e00202-18. doi: 10.1128/mBio.00202-18 29691333 PMC5915736

[B99] Takahashi N. Kariu T. Hayashi M. Kishimoto T. Ogasawara C. Fukuyama A.. (2023). The effect of *Porphyromonas gingivalis* on inflammatory pathology in normal or COPD airway’, in *Airway cell biology and immunopathology* . Eur. Respir. Soc. 62, PA4667. doi: 10.1183/13993003.congress-2023.PA4667

[B100] Tamai R. Sugamata M. Kiyoura Y. (2011). *Candida albicans* enhances invasion of human gingival epithelial cells and gingival fibroblasts by *Porphyromonas gingivalis* . Microbial Pathogenesis 51, 250–254. doi: 10.1016/j.micpath.2011.06.009 21742026

[B101] Travis J. Pike R. Imamura T. Potempa J. (1997). *Porphyromonas gingivalis* proteinases as virulence factors in the development of periodontitis. J. Periodontal Res. 32, 120–125. doi: 10.1111/j.1600-0765.1997.tb01392.x 9085221

[B102] Tribble G. D. Lamont R. J. (2010). Bacterial invasion of epithelial cells and spreading in periodontal tissue. Periodontology 2000 52, 68–83. doi: 10.1111/j.1600-0757.2009.00323.x 20017796 PMC3647226

[B103] Van Der Post S. Subramani D. B. Bacükström M. Johansson M. E. V. Vester-Christensen M. B. Mandel U.. (2013). Site-specific O-glycosylation on the MUC2 mucin protein inhibits cleavage by the *Porphyromonas gingivalis* secreted cysteine protease (RgpB). J. Biol. Chem. 288, 14636–14646. doi: 10.1074/jbc.M113.459479 23546879 PMC3656315

[B104] Wegner N. Wait R. Sroka A. Eick S. Nguyen K. A. Lundberg K.. (2010). Peptidylarginine deiminase from *Porphyromonas gingivalis* citrullinates human fibrinogen and α-enolase: Implications for autoimmunity in rheumatoid Arthritis. Arthritis Rheumatism 62, 2662–2672. doi: 10.1002/art.27552 20506214 PMC2941529

[B105] Yilmaz Ö. (2008). The chronicles of *Porphyromonas gingivalis*: The microbium, the human oral epithelium and their interplay. Microbiology 154, 2897–2903. doi: 10.1099/mic.0.2008/021220-0 18832296 PMC2639765

[B106] Zarrinfar H. Kaboli S. Dolatabadi S. Mohammadi R. (2016). Rapid detection of *Candida* sp*ecies* in bronchoalveolar lavage fluid from patients with pulmonary symptoms. Braz. J. Microbiol. 47, 172–176. doi: 10.1016/j.bjm.2015.02.001 26887241 PMC4822774

[B107] Zhang J. Xie M. Huang X. Chen G. Yin Y. Lu X.. (2021). The effects of *porphyromonas gingivalis* on atherosclerosis-related cells. Front. Immunol. 12. doi: 10.3389/fimmu.2021.766560 PMC8734595 35003080

[B108] Zhao Q. Wang X. Liu W. Tian H. Yang H. Wang Z.. (2024). *Porphyromonas gingivalis* inducing autophagy-related biological dysfunction in alveolar epithelial cells: an *in vitro* study. BMC Oral. Health 24, 1478. doi: 10.1186/s12903-024-05253-y 39639253 PMC11619664

