# Supplementary material for: Synergistic effects of Candida albicans and Porphyromonas gingivalis biofilms on epithelial barrier function in a 3D aspiration pneumonia model
Source: Front Cell Infect Microbiol. 2025 Mar 7;15:1552395. doi: 10.3389/fcimb.2025.1552395 (PMC11925950; doi:10.3389/fcimb.2025.1552395)
Supplement: Supplementary file 1 [file DataSheet1.pdf]

Synergistic effects of *Candida albicans* and *Porphyromonas gingivalis* biofilm on epithelial barrier function in a 3D aspiration pneumonia model

Ewelina Wronowska[1], Ibeth Guevara-Lora[1], Aleksandra Brankiewicz[1,2], Grazyna Bras[1], Marcin Zawrotniak[1], Dorota Satala[1], Justyna Karkowska-Kuleta[1], Joanna Budziaszek[3], Joanna Koziel[3], Maria Rapala-Kozik[1*]

[1]Department of Comparative Biochemistry and Bioanalytics, Faculty of Biochemistry, Biophysics and Biotechnology, Jagiellonian University in Krakow, Krakow, Poland

[2]Doctoral School of Exact and Natural Sciences, Faculty of Biochemistry, Biophysics and Biotechnology, Jagiellonian University, Gronostajowa 7, 30-387 Kraków, Poland

[3]Department of Microbiology, Faculty of Biochemistry, Biophysics and Biotechnology, Jagiellonian University, Gronostajowa 7, 30-387 Kraków, Poland

*** Correspondence:**
Maria Rapala-Kozik
maria.rapala-kozik@uj.edu.pl

Supplementary Material

1. Analysis of data acquired from mitochondrial activity measurements in human epithelial cells cultured in the BIOFILM model.

To assess mitochondrial activity in BEAS-2B epithelial cells after 24 hours of infection with single- or dual-species biofilms, MitoTracker™ Orange CMTMRos was used. This dye selectively accumulates in active mitochondria. However, since MitoTracker™ Orange CMTMRos localizes in both *Candida albicans* and BEAS-2B cells (Supplementary Figure 1), fungal cells were additionally stained with Calcofluor White Stain, which emits fluorescence in the blue channel. Imaging was performed using a Leica Stellaris 5 confocal microscope, capturing a series of optical sections as a Z-stack. Fluorescence analysis was then conducted for each Z-plane to accurately exclude fungal regions from the mitochondrial activity assessment of host cells. Based on the calcofluor signal, areas containing *C. albicans* were identified and excluded from fluorescence measurements in the red channel for MitoTracker™ Orange CMTMRos. The fluorescence signal obtained through this method originated from active mitochondria in human cells, and values from three different areas were averaged and quantified, as shown in Figure 1A. All analyses were performed using Fiji software (ImageJ, version 1.54f, USA).

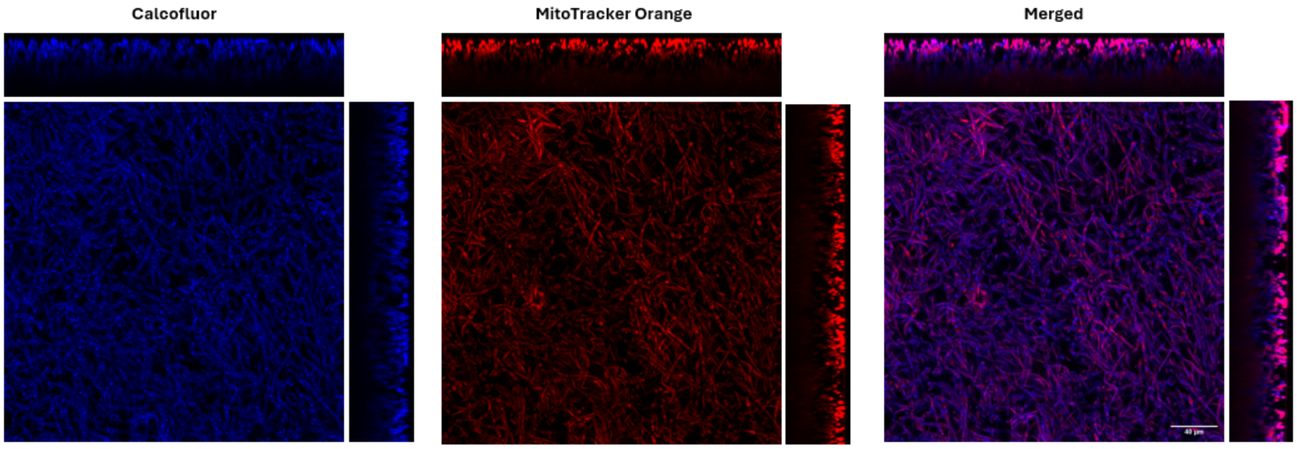

Supplementary Figure 1. The representative orthogonal view for a 24-hour *C. albicans* biofilm formed on BEAS-2B cells. The sample was stained with Calcofluor White Stain (blue) at a dilution of 1:1000 and MitoTracker™ Orange CMTMRos (red) at a concentration of 1µM and then imaged with a Leica Stellaris 5 confocal microscope.
